# The carboxy‐terminal tail of GLR3.3 is essential for wound‐response electrical signaling

**DOI:** 10.1111/nph.18475

**Published:** 2022-09-28

**Authors:** Qian Wu, Stéphanie Stolz, Archana Kumari, Edward E. Farmer

**Affiliations:** ^1^ Shenzhen Branch, Guangdong Laboratory of Lingnan Modern Agriculture, Genome Analysis Laboratory of the Ministry of Agriculture and Rural Affairs Agricultural Genomics Institute at Shenzhen, Chinese Academy of Agricultural Sciences Shenzhen 518120 China; ^2^ Department of Plant Molecular Biology, Biophore University of Lausanne Lausanne CH‐1015 Switzerland

**Keywords:** Arabidopsis, defense, electrical signaling, glutamate receptor, herbivory, jasmonate, phloem, wounding

## Abstract

- *Arabidopsis* Clade 3 *GLUTAMATE RECEPTOR‐LIKEs* (*GLR*s) are primary players in wound‐induced systemic signaling. Previous studies focused on dissecting their ligand‐activated channel properties involving extracellular and membrane‐related domains. Here, we report that the carboxy‐terminal tails (C‐tails) of GLRs contain key elements controlling their function in wound signaling.
- GLR3.3 without its C‐tail failed to rescue the *glr3.3a* mutant. We carried out a yeast two‐hybrid screen to identify the C‐tail interactors. We performed functional studies of the interactor by measuring electrical signals and defense responses. Then we mapped their binding sites and evaluated the impact of the sites on GLR functions.
- IMPAIRED SUCROSE INDUCTION 1 (ISI1) interacted with GLR3.3. Enhanced electrical activity was detected in reduced function *isi1* mutants in a GLR3.3‐dependent manner. *isi1* mutants were slightly more resistant to insect feeding than the wild‐type. Furthermore, a triresidue motif RFL in the GLR3.3 C‐tail binds to ISI1 in yeast. Finally, we demonstrated that FL residues were conserved across GLRs and functionally required.
- Our study provides new insights into the functions of GLR C‐tails, reveals parallels with the ionotropic glutamate receptor regulation in animal cells, and may enable rational design of strategies to engineer GLRs for future practical applications.

## Introduction

Clade 3 *GLUTAMATE RECEPTOR‐LIKE* (*GLR*) genes encode ancient ion channels that play diverse roles in both sporophytes and gametophytes throughout the plant kingdom (De Bortoli *et al*., 2016; Wudick *et al*., 2018a). For example, these proteins function in sperm chemotaxis in a basal land plant lineage represented by the moss *Physcomitrella* (Ortiz‐Ramirez *et al*., 2017). In the angiosperm *Arabidopsis*, clade 3 GLRs function in pollen tube growth (Michard *et al*., 2011; Wudick *et al*., 2018b), lateral root development (Vincill *et al*., 2013), and plant regeneration (Hernandez‐Coronado *et al*., 2022). One of the seven clade 3 GLRs in *Arabidopsis*, GLR3.3, stands out for its roles in plant defense. First, *glr3.3* mutations reduce the resistance of this plant to the fungus *Hyaloperonospora* (Manzoor *et al*., 2013). Second, along with GLR3.1, ‑3.2, and ‑3.6, GLR3.3 functions in wound‐response electrical signaling, leading to activation of the jasmonate pathway (Mousavi *et al*., 2013; Nguyen *et al*., 2018; Toyota *et al*., 2018). The jasmonate pathway (Browse, 2009) underlies defense against many insects (Erb & Reymond, 2019; J. Wang *et al*., 2019).

Plant GLRs and mammalian ionotropic glutamate receptors (iGluRs) are homologues. Exemplified by the recently resolved GLR3.4 structure (Green *et al*., 2021), GLRs are multi‐membrane‐spanning proteins possessing a large extracellular amino‐terminal domain (ATD), one ligand‐binding domain (LBD), three transmembrane domains, one pore region, and a cytoplasmic carboxyl‐terminal domain (CTD) (Weiland *et al*., 2015; Grenzi *et al*., 2021). The ATD, LBD, and the membrane‐associated domains of the homologues are crucial in determining their ligand‐activated channel properties (Wudick *et al*., 2018a). In the case of GLR3.3, its LBD was recently resolved in combination with amino acids (Alfieri *et al*., 2020), confirming the broad gating‐ligand specificity as was also reported *in vivo* (Qi *et al*., 2006). Nevertheless, the researchers failed to identify any defective ligand binding sites in GLR3.3 LBD due to the insolubility of the mutant proteins (Alfieri *et al*., 2020). Overall, despite growing structural and biochemical characterizations of GLR3.3, little information for any functional sites in the protein is available to date. With regard to the CTD of GLRs, how it contributes to GLR functions remains a mystery. The CTD sequences of mammalian iGluRs and plant GLRs diverged during evolution (Wudick *et al*., 2018a). Plant GLR carboxy‐terminal tails (C‐tails) have *c*. 80 amino acids that are theoretically long enough to associate physically with other proteins. Indeed, the C‐tails of all clade 3 GLRs contain putative endoplasmic reticulum retention signals (Wudick *et al*., 2018a), and the C‐tails of GLR3.4 and GLR3.7 bind to 14‐3‐3 proteins (Chang *et al*., 2009; P. H. Wang *et al*., 2019). However, how these and other potential C‐tail features influence GLR functions remains to be explored.

In this study, we report that GLR C‐tails contain key elements that are required for their function in wound signaling. We found that the C‐tail interacts with a protein called Impaired Sucrose Induction 1 (ISI1). We further demonstrated that ISI1 plays a role in wound‐induced responses. Moreover, by employing a mutagenesis‐based mapping strategy, we dissected the binding sites of ISI1 in GLR3.3 C‐tail and then confirmed their essential functions in plants. Finally, we extended our study to all three GLR C‐tails and discovered two conserved residues are required for GLR *in vivo* functions. Our work reveals for the first time how GLR C‐tails shape their functions in plants. We hypothesize that, despite sequence diversities, the C‐tails of animal and plant GLRs parallel in exerting regulatory roles in affecting their functions.

## Materials and Methods

### Plant materials and growth conditions

*Arabidopsis thaliana* accession Columbia (Col‐0) was used as wild‐type (WT) and is the background of all the mutants investigated in this study. The transfer DNA (T‐DNA) insertion lines *glr3.3a* (SALK_099757) and *glr3.1a* (SALK_063873) were reported in Mousavi *et al*. (2013). *isi1‐2* (SALK_014032), *isi1‐3* (SALK_045849), and *IMPORTIN SUBUNIT alpha‐2* (*impa2*)*‐1* (SALK_017914) were from the Nottingham *Arabidopsis* Stock Centre. Primers used for genotyping *ISI1* and *IMPA2* alleles are listed in Table S1. To work with 5‐wk‐old plants, seeds were sown individually in 7 cm diameter pots. Plants were stratified at 4°C for 2 d in the dark before transferring them to the growth room at 21°C under 150 μmol m^−2^ s^−1^ light (10 h : 14 h, light : dark, 70% humidity).

### Generation of the transgenic plants

To make *GLR3.3*_*pro*_ *:GLR3.3ΔCT‐mVENUS/glr3.3a* plants, the *GLR3.3*_*pro*_ *:GLR3.3ΔCT* genomic fragment was amplified from the plasmid pUC57‐*GLR3.3*_*pro*_ *:GLR3.3* genomic clone published in Nguyen *et al*. (2018) and cloned into pUC57‐L4‐*Kpn1*/*Xma1*‐R1 via *Kpn1* and *Xma1* sites. Double Gateway cloning was carried out to combine GLR3.3_pro_:GLR3.3ΔCT in pUC57 and pEN‐L1‐mVENUS‐L2 with the destination vector pH7m24GW. In this study, to introduce point mutations into GLRs for transgene, the corresponding residues were all converted to alanine. Specifically, to introduce desired point mutations into the GLR3.3_*pro*_:GLR3.3‐mVENUS or GLR3.1_*pro*_:GLR3.1‐mVENUS fusions, PCRs were performed with mutagenic overlapping primers designed with the QuikChange Primer Design tool (https://www.agilent.com/store/primerDesignProgram.jsp) to amplify the entire plasmid pUC57‐*GLR*_*pro*_ *:GLR* genomic. Primers used to generate the mutations are listed in Table S1. Together with pEN‐L1‐mVENUS‐L2, all the resulting pUC57‐GLR3.3_*pro*_:GLR3.3 clones with the indicated mutations were recombined with the destination vector pH7m24GW, respectively, to generate binary expression vectors and then transformed into *glr3.3a* mutant plants. T_1_ seeds were selected by adding 25 mg ml^−1^ hygromycin to the half‐strength Murashige & Skoog plates. In the case of GLR3.1_*pro*_:GLR3.1 with point mutations, the destination vector was pEDO097pFR7m24GW (Shimada *et al*., 2010). The resulting T_1_ plants were selected based on seed coat fluorescence using an MZ16 FA microscope (Leica, Wetzlar, Germany). T_3_ plants that were homozygous for the antibiotic were used for studies. At least two independent transgenic lines were used for the experiments in this work.

To generate *ISI1*_*pro*_ *:ISI1‐mCherry/isi1‐2* plants, the full‐length *ISI1* genomic sequence spanning the 1175 bp promoter region and the gene (*ISI1*_*pro*_ *:ISI1*) was amplified and subsequently cloned into pUC57‐L4‐*Kpn1*/*Xma1*‐R1 by digestion and ligation. Primers used for cloning *ISI1* are listed in Table S1. To obtain an *ISI1*_*pro*_ *:ISI1‐mCherry* expression clone, *ISI1*_*pro*_ *:ISI1* in pUC57, *mCherry* coding sequence in L1‐pDONOR221‐L2, and the destination vector pEDO097pFR7m24GW were combined by double Gateway cloning. Transgenic plants were obtained by dipping *isi1‐2* plants with *Agrobacterium* carrying the corresponding vector. Similarly, translational reporter plants *IS11*_*pro*_ *:ISI1‐GUSPlus* were made. In the latter case, mCherry in L1‐pDONOR221‐L2 was replaced by pEN‐L1‐GUS*Plus*‐L2 for the final recombination, and Col‐0 plants were transformed. The resulting T_1_ plants were selected based on seed coat fluorescence. T_3_ plants were used for all the analysis in this work.

### Surface potential measurements

Protocols for monitoring slow wave potentials (SWPs) were detailed elsewhere (Mousavi *et al*., 2013, 2014; Nguyen *et al*., 2018). Briefly, silver/silver chloride electrodes were placed on the petioles of both leaves 8 (L8) and 13 (L13) from 5‐wk‐old plants. The connection between electrodes and leaf surface was maintained by adding one drop of 10 mM potassium chloride in 0.5% (w/v) agar. A reference electrode was placed in the soil. For wounding, 50–60% of the apical lamina surface distal to the rosette center of L8 was crushed with a plastic forceps. Electrical signals were recorded from both leaves at 100 Hz using LabScribe3 (iWorx System Inc., Dover, NH, USA) software. Amplitudes and durations of the measured electrical signals were analyzed as described in Mousavi *et al*. (2013).

### Visualization of protein subcellular localization by confocal microscopy

To observe the subcellular localization of GLR3.3‐mVENUS and its derivatives, vein samples were prepared from expanded leaves of 5‐wk‐old plants. Vein extraction was performed following the description in Kurenda & Farmer (2018). Isolated veins were immediately fixed with 4% paraformaldehyde (PFA) solution for 1 h with gentle shaking and then subjected to ClearSee treatment (Ursache *et al*., 2018) for 2 d. Refreshing the ClearSee solution was necessary to get sufficiently cleared samples. To stain samples, 0.1% (w/v) Calcofluor‐white was added into the ClearSee solution, and the samples were stained for 15 min. Then the samples were washed twice with ClearSee solution before observation. All the samples were visualized with an SP8 microscope (Leica Microsystems CMS GmbH, Mannheim, Germany). Sequential scanning mode was used to avoid interference between channels. mVENUS was excited at 514 nm and detected in the range 520–540 nm. In most cases, Chl autofluorescence still remained and was detected in an emission window of 650–700 nm. Calcofluor‐white was imaged with 405 nm excitation and 430–460 nm emission.

### Yeast two‐hybrid assay and Western blotting

A yeast two‐hybrid (Y2H)‐based screen was carried out with the ULTImate Y2H platform (Hybrigenics Services, Evry, France). For this, the C‐terminal tail of GLR3.3 (850–933 amino acids (aa)) was constructed into pB27 vector (N‐LexA‐bait‐C fusion) as a bait to screen against the prey complementary DNA (cDNA) library made from *Arabidopsis* rosette leaves. A total of 146 millions interactions were analyzed, and 84 clones were further processed. ISI1 and IMPA2 appeared as two of the clones with very high confidence in the interactions. To test the interactions, the C‐tails of GLRs and their variants were cloned into the commercial pGBKT7 vector as baits. IMPA2 and ISI1 and its variants were each inserted into pGADT7‐Rec vectors as preys. The primers used for cloning and generating the indicative mutations are available in Table S1. GLR3.3 sequences with single F‐to‐A and L‐to‐A mutations, GLR3.1 with T‐to‐R conversion, and GLR3.6 with S‐to‐R conversion were all synthesized (Azenta, Suzhou, China). For the other point mutations, the corresponding residues were all converted to alanine in the yeast assays. Each of the bait and prey construct pairs were co‐transformed into the yeast strain AH109 and the interactions were analyzed by growing transformants on selective medium as described in Wu *et al*. (2016). For total yeast protein extraction, the yeast cells were harvested at OD_600_ = 0.8–1.0 and then resuspended with 1 × TBS buffer. The cells were then disrupted by beads beating at 30 Hz for 1 min, with the procedure being repeated five times. Then 4× protein loading buffer was added to the cell lysates, followed by boiling for 5 min at 100°C. The samples were centrifuged and the supernatants were loaded for Western blot analysis. α‐HA (1 : 5000; Sigma‐Aldrich, St Louis, MO, USA) and α‐Myc (1 : 1000; Proteintech, Wuhan, China) antibodies were used to detect AD‑ and BD‐tagged proteins, respectively.

### Firefly luciferase complementation imaging assay

The full‐length *GLR3.3* cDNA (Wudick *et al*., 2018b) was fused upstream of the N‐terminal part of *Luciferase* (*nLUC*) in the pCAMBIA1300‐nLUC vector by infusion cloning. Similarly the C‐tail of *GLR3.3* was intro duced by conventional cloning via sites Kpn1 and Sal1. *ISI1* cDNA was fused downstream of the C‐terminal part of *Luciferase* (*cLUC*) in the pCAMBIA1300‐cLUC vector. The primers used for the noted constructions are listed in Table S1. Each of the constructs for the assay was transferred into *Agrobacterium* strain GV3101. To determine the interactions of full‐length or the C‐tail GLR3.3 with ISI1 in *Nicotiana benthamiana* leaves, *Agrobacterium* harboring the indicated constructs were resuspended in the infiltration buffer containing 10 mM magnesium chloride, 10 mM MES, 0.5 g l^−1^ glucose, and 150 μM acetosyringone to a final concentration of OD_600_ = 0.5. Then, equal volumes of different combinations were mixed and coinfiltrated into the abaxial face of tobacco leaves using a needleless syringe. Plants were then kept for 24 h in the dark before transferring them to light for another 24–48 h. To facilitate and observe the luminescence brought about by the interactions of the proteins, the *N. benthamiana* leaves were fully infiltrated with 0.1 mg ml^−1^ luciferin and placed in the dark for 5 min before CCD imaging. LUC activity was determined using an IVIS Lumina III In Vivo Imaging System (PerkinElmer, Richmond, CA, USA). The exposure time was from 1 to 5 min depending on the signal intensity.

### RNA extraction and reverse transcription quantitative PCR

To detect the expression of wound‐response marker gene *JAZ10*, L8 from 5‐wk‐old plants was wounded. L13 samples were harvested 1 h post‐wounding and used for RNA extraction. The procedures for cDNA reverse transcription (RT) and quantitative PCR (qPCR) were described in Gfeller *et al*. (2011). qPCR data were normalized to the reference gene *ubiquitin‐conjugating enzyme 21* (*UBC21*). Primers for *UBC21* and *JAZ10* were used previously (Mousavi *et al*., 2013). Primers to detect transcripts generated from the 5′ end and 3′end of *ISI1* as shown in Fig. S2a (see later) are listed in Table S1.

### Electrical penetration graph recordings

Electrical penetration graph (EPG) was employed to study sieve elements‐specific electrical signals. The experimental setup and data analysis were detailed previously (Salvador‐Recatala *et al*., 2014; Kumari *et al*., 2019).

### Insect bioassays

Details for preparing the insects were described previously (Fotouhi *et al*., 2022). For the bioassay, 11 pots of 5‐wk‐old plants were placed in Plexiglass boxes (28.5 × 19 × 19 cm^3^). Four freshly hatched *Spodoptera littoralis* larvae were gently placed on the rosette center of each plant with a soft brush. After feeding for up to 10 d, the caterpillars were collected and weighed from individual boxes. The caterpillar mass from each box was considered as one replicate. The average weight from four replicates and the total numbers of the surviving caterpillars were recorded.

### β‐Glucuronidase staining and sectioning

Three‐week‐old *IS11*_*pro*_ *:ISI1‐GUSPlus/Col‐0* plants were excised for β‐glucuronidase (GUS) staining. The expanded leaves were immediately fixed after excision in 90% acetone on ice for 1 h, followed by twice washing with 50 mM sodium phosphate buffer (pH 7.4). Then the plants were stained by adding staining solution (10 mM EDTA disodium salt, 50 mM sodium phosphate buffer, 1 mM potassium ferrocyanide, 1 mM potassium ferricyanide, 0.1% (v/v) Triton X‐100, 0.5 mg ml^−1^ X‐Gluc (pH 7.2)) and subjected to vacuum infiltration for 30 min. After incubating at 37°C in the dark for 6 h, the plants were washed with 50 mM sodium phosphate buffer and then cleared with 70% (v/v) ethanol. Images of plants were taken with a VHX‐6000 digital microscope (Keyence, Osaka, Japan). To study the detailed expression pattern of ISI1 at cellular level, the petioles of the expanded leaves were further fixed in glutaradehyde/formaldehyde/50 mM sodium phosphate (pH 7.2) 2 : 5 : 43 (v/v/v) for 30 min and then dehydrated with ethanol gradients (10%, 30%, 50%, 70%, 90%, and twice absolute) for 30 min in each concentration. Afterwards, they were embedded in Technovit 7100 resin (Haslab GmbH, Ostermundigen, Switzerland) according to the manufacturer's instructions. Transversal sections (5 μM thick) were cut on an RM2255 microtome (Leica). The sections were mounted in 40% (v/v) glycerol and then imaged with a Leica DM5500 microscope.

### Plant protein extraction and Western blotting

Approximately 100 mg midveins from 5‐ to 6‐wk‐old *ISI1*_*pro*_ *:ISI1‐mCherry/isi1‐2* plants were harvested according to protocol in Kurenda & Farmer (2018) and frozen for protein extraction. The lamina parts of the leaves after midvein removal were also collected for analysis. Frozen tissues were ground to fine powder with a TissueLyser (Qiagen, Hilden, Germany). Proteins were extracted with lysis buffer (50 mM Tris hydrochloride, pH 7.5, 150 mM sodium chloride, 0.1% (v/v) Nonidet P‐40) plus plant‐specific protease inhibitor cocktail (Sigma‐Aldrich Chemie GmbH, Buchs SG, Switzerland). After centrifuging at 16 200 ***g*** for 15 min at 4°C, the supernatants were collected. The protein samples were prepared by mixing the supernatant with 4× sodium dodecyl sulfate protein loading buffer, followed by incubating for 5 min at 95°C, and then separated by running a 4%–12% (v/v) polyacrylamide gradient ExpressPlus™ Bis‐Tris gel (GenScript, Piscataway, NJ, USA). Immunoblotting was employed to detect the ISI1‐mCherry fusion protein by using anti‐mCherry antibody (ab167453, polyclonal; Abcam, Cambridge, UK). Ponceau staining was used to assess correct gel loading.

### Structural prediction and analysis

The structural model of GLR3.3 was predicted using the AlphaFold Protein Structure Database (https://alphafold.ebi.ac.uk) (Jumper *et al*., 2021; Varadi *et al*., 2022). PyMOL (PyMOL Molecular Graphics System v.2.0; Schrödinger, LLC, New York, NY, USA) was used to visualize the protein structure and the labels. Phyre2 (Protein Homology/analogY Recognition Engine v.2.0, http://www.sbg.bio.ic.ac.uk/phyre2/html/page.cgi?id=index) (Kelley *et al*., 2015) was used to predict the secondary structure of GLR3.3 based on homology modeling. Normal modeling mode was used.

### Sequence alignment

The C‐tail protein sequences of GLR3.3 (850–933 aa), GLR3.1 (853–925 aa), and GLR3.6 (843–903 aa) were retrieved from UniprotKB and subjected to a multiple sequence alignment using Clustal Omega (https://www.ebi.ac.uk/Tools/msa/clustalo/) (Madeira *et al*., 2019). The aligned result was graphed with ESPript (https://espript.ibcp.fr/ESPript/ESPript/) (Robert & Gouet, 2014).

## Results

### The carboxy‐terminal tail of GLR3.3 is required for its function

**Fig. 1:**
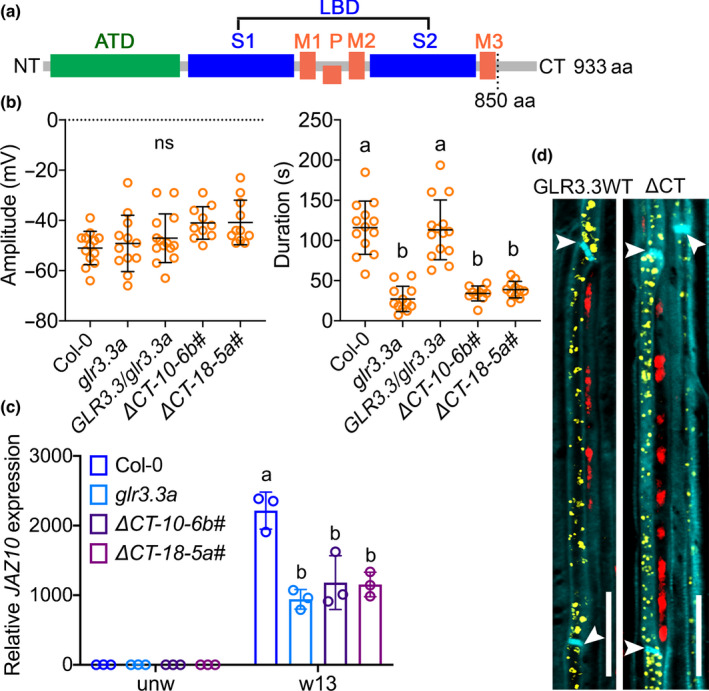
Deletion of the carboxy‐terminal tail (C‐tail) of GLUTAMATE RECEPTOR‐LIKE (GLR)3.3 impairs its function in wound‐induced electrical signaling and defense gene activation, but not subcellular distribution in Arabidopsis. (a) Schematic diagram showing GLR architecture. ATD, amino‐terminal domain; LBD, ligand binding domain; S1 and S2, segment 1 and segment 2; M1 to M3, membrane‐spanning domain 1 to 3; P, pore region; CT, C‐tail. (b) Amplitude and duration of surface potential changes on leaf 13 after wounding leaf 8. Wild‐type (WT), *glr3.3a* mutants, *glr3.3* complemented plants (*GLR3.3/glr3.3a*), and two independent lines for the C‐tail deletion plants were measured. The orange circles represent individual plants. *n* = 10–14. The horizontal bars indicate the mean values. Error bars show SD. The different letters indicate significant differences (*P* < 0.01) after one‐way ANOVA. ns, not significant. ΔCT is the deletion of the C‐tail from residues 850 to 933. (c) *JAZ10* expression levels in unwounded leaf 8 and distal leaf 13 after wounding leaf 8. Data shown are means ± SD. Each colored circle represents one biological replicate. *n* = 3. The different letters indicate significant differences (*P* < 0.01) after two‐way ANOVA. (d) Subcellular localization of full‐length GLR3.3 protein and its C‐tail deletion. Midveins from plants expressing GLR3.3‐mVENUS (GLR3.3WT) or GLR3.3 ΔCT–mVENUS (ΔCT) fusions under the *GLR3.3* promoter were extracted and mVENUS was localized by confocal microscopy. Yellow is signal from mVENUS fusion proteins. Red is chlorophyll autofluorescence from companion cells. Cyan marks outlines of the cells. Arrowheads indicate the positions of sieve plates. Images were taken with the same parameters. Bar, 20 μm.

GLR3.3 contains a putative intracellular C‐tail of 83 aa from position 850 to 933 aa (Fig. 1a). To determine if this tail of GLR3.3 was essential for function, we generated GLR3.3 lacking its C‐tail. The truncated GLR3.3 was then fused with mVENUS and transformed into the *glr3.3a* mutant background in order to investigate its ability to rescue the *glr3.3a* mutant phenotype. Upon wounding L8, slow wave potentials (SWPs) in both the wounded (L8) and the distal connected leaves (L13) were measured in two independent lines of the C‐tail‐deleted plants, along with WT, a *glr3.3a* mutant, and the GLR3.3 complemented line from a previous study (*GLR3.3pro:GLR3.3‐mVENUS#2.3*, named as *GLR3.3/glr3.3a* in this study) (Nguyen *et al*., 2018) as controls. L8 signals varied slightly in terms of the amplitudes and durations among the aforementioned genotypes (Fig. S1). No substantial differences were found in the amplitude of the L13 SWPs recorded from all the genotypes (Fig. 1b, left panel). However, compared with the WT and the complemented plants (*GLR3.3/glr3.3a*), the lines that express GLR3.3 lacking its C‐tail (*ΔCT‐10‐6b#* and *ΔCT‐18‐5a#*) failed to rescue the *glr3.3a* knockout phenotype in propagating SWPs and showed strongly reduced, *glr3.3a* mutant‐like duration of L13 SWPs (Figs 1b, S1a). Next, to assess if the defect in propagating SWPs between leaves alters the systemic activation of the jasmonate pathway, we measured the expression of the wound‐induced jasmonate pathway marker gene *JAZ10* in distal connected L13 after wounding L8. In these experiments, *JAZ10* expression was similarly attenuated in the distal leaves (L13) of C‐tail variants as in the *glr3.3a* mutants (Fig. 1c). Together, these results showed that the short C‐tail of GLR3.3 is required for its functions in wound signaling.

We next investigated whether deletion of the GLR3.3 C‐tail altered its cellular distribution. Previously, it was reported that the major pool of GLR3.3‐mVENUS protein co‐localized with an endoplasmic reticulum (ER) marker in sieve elements (Nguyen *et al*., 2018). Using the same line as control (Fig. 1d, GLR3.3WT), a similar mVENUS distribution pattern was obtained in plants expressing the GLR3.3 variant (Fig. 1d, ΔCT). Given that neither the expression nor the localization of GLR3.3 was impaired, the inability of GLR3.3 C‐tail deletion to rescue the *glr3.3a* phenotype indicates that the C‐tail of GLR3.3 contains motifs that are functionally important to preserve GLR protein activity or to interact with other regulators.

### ISI1 binds to GLR3.3 carboxy‐terminal tail *in vivo*

**Fig. 2:**
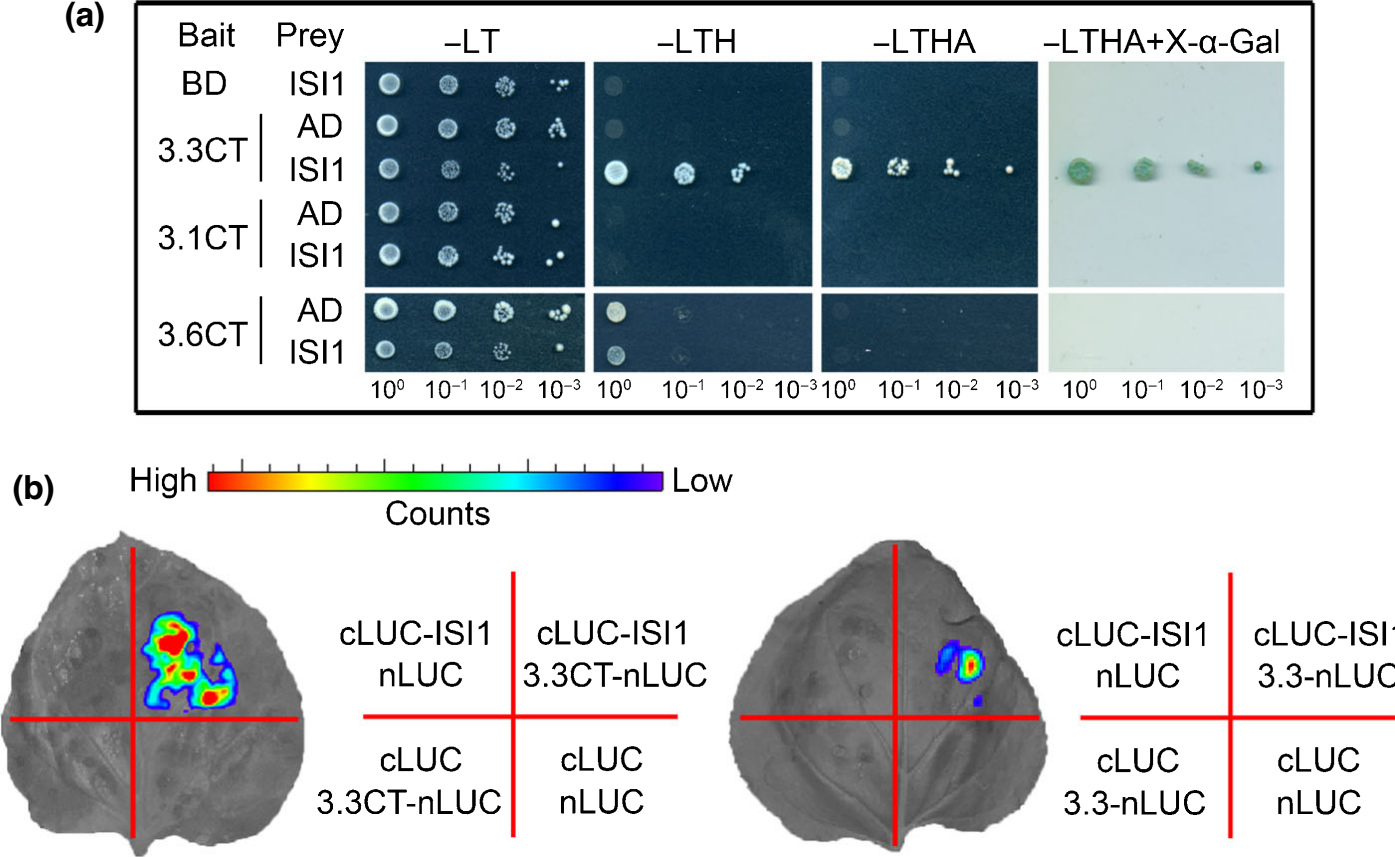
IMPAIRED SUCROSE INDUCTION 1 (ISI1) interacts with the GLUTAMATE RECEPTOR‐LIKE (GLR)3.3 carboxy‐terminal tail (C‐tail) *in vivo*. (a) ISI1 interacts specifically with the C‐tail (CT) of GLR3.3, but not GLR3.1 and GLR3.6 in yeast two‐hybrid assays. AD‐fused ISI1 was co‐transformed with BD‐fused C‐tail of GLR3.3, GLR3.1, or GLR3.6. Empty AD or BD vectors were included as negative controls. The interactions were tested by growing yeast cells on different selective media. Photographs were taken after 3 d for yeast grown on Leu‐Trp‐ (−LT)/yeast nitrogen base medium or 5 d for the other yeast groups on selective Leu‐Trp‐His‐ (−LTH), Leu‐Trp‐His‐Ade (−LTHA), and −LTHA plus X‐α‐Gal media. (b) Firefly luciferase (LUC) complementation imaging assay showing the interactions of ISI1 with the C‐tail and the full‐length GLR3.3 proteins. Construct pairs as indicated in the right panel of (b) were coexpressed in *Nicotiana benthamiana* leaves. The experiments were performed three times with consistent results. Representative pictures are shown for the interactions. The color scale reflects LUC activity.

To test the hypothesis that the C‐tail of GLR3.3 is likely involved in binding other regulators, a Y2H screen was conducted using the GLR3.3 C‐tail as bait. The complete list of all the candidate genes from the screen is available in Table S2. *ISI1* (AT4G27750) and *IMPA2* (AT4G16143) were the candidates with the highest confidence for interactions (Table S2). ISI1 was able to bind to GLR3.3CT but not to either the C‐tail of GLR3.1 (3.1CT) or GLR3.6 (3.6CT) (Fig. 2a). The *in vivo* interaction between GLR3.3 and ISI1 was further confirmed in the firefly luciferase complementation imaging assay. In contrast to the negative controls, ISI1 interacted with both the C‐tail and the full‐length GLR3.3 proteins (Fig. 2b).

### Vasculature‐associated *ISI1* is involved in wound‐induced long‐distance signaling

**Fig. 3:**
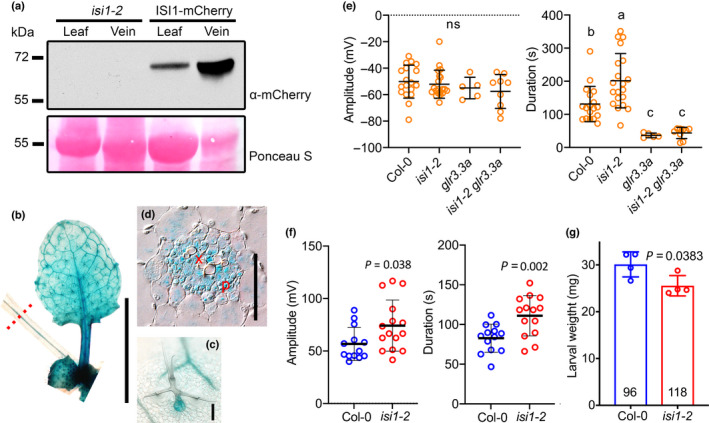
Vasculature‐associated *IMPAIRED SUCROSE INDUCTION 1* (*ISI1*) affects surface and sieve‐elements‐specific electrical signal durations, as well as defense against insects in Arabidopsis. (a) Western blot for ISI‐mCherry fusion proteins from midveins or lamina tissues from which the midveins were removed. Proteins were extracted from 5‐wk‐old *ISI1*_*pro*_ *:ISI1‐mCherry* plants and detected with mCherry antibodies. Ponceau S staining was used to assure comparable loading of each well. (b–d) β‐Glucuronidase (GUS) staining for ISI protein expression pattern from 3‐wk‐old *ISI1*_*pro*_ *:ISI1*_*genomic*_ *‐GUS* plants. (b) GUS activity for a rosette leaf; bar, 1 cm. (c) Stained trichome; bar, 100 μm. (d) Transversal section of the petiole at the position indicated by the red dashed line in (b). Bar, 50 μm. P, phloem region; X, xylem region. (e) Amplitude (left panel) and duration (right panel) of surface potential changes on leaf 13 after wounding leaf 8 recorded from different genotypes. Colored circles indicate individual biological replicates. *n* = 5–20. The horizontal bars indicate the mean values. Error bars show SD. The different letters indicate significant differences after one‐way ANOVA. ns, not significant. (f) Amplitudes (left panel) and durations (right panel) for sieve‐elements‐specific electrical signals detected with aphid electrodes from leaf 13 after wounding leaf 8. Colored circles shown are measurements from individual plants. *n* = 13–14. The horizontal bars indicate the mean values. Error bars show SD. *P* values were calculated with two‐tailed Student's *t*‐tests. (g) Larval weight gain on *isi1‐2* vs wild‐type plants after feeding for 11 d. Four biological replicates were analyzed and are represented by the colored circles. Each replicate is the average larval weight from 44 larvae that were initially placed in one box. Numbers in each bar indicate how many larvae survived at the end of the experiment. Data shown are means ± SD. *P*‐values were calculated with two‐tailed Student's *t*‐tests. ns, not significant.

To dissect the roles of *ISI1*, we obtained two T‐DNA insertion alleles, *isi1‐2* (Salk_014032) (Rook *et al*., 2006) and *isi1‐3* (Salk_045849) (Fig. S2a,b). First, we determined the expression pattern of ISI1. We created transgenic plants harboring an ISI1‐encoding genomic fragment in fusion with a fluorescent mCherry tag in the *isi1‐2* mutant background. In the Western blotting analysis, more ISI1‐mCherry fusion protein was detected in midvein samples than in the extracts from leaf lamina without midvein (Fig. 3a). Similarly, in 3‐wk‐old rosettes expressing *IS11*_*pro*_ *:ISI1‐GUSPlus* in WT background, ISI1‐GUS was distributed in the whole leaf, including the trichomes (Fig. 3b,c). However, in a transversally sectioned petiole, GUS staining was detected in both phloem and xylem regions and appeared to be more abundant in the vascular bundles than in the surrounding cells (Fig. 3d). In the phloem region, ISI1 localized to cytoplasm and nucleus in companion cells (Fig. S2c), consistent with its subcellular localization in root cells reported by Rook *et al*. (2006).

Next, wound‐induced L8 and L13 SWPs were measured in the *isi1* T‐DNA insertion mutants. In the leaves distal to wounds (L13), prolonged durations of the surface potential were detected in both *isi1* mutant lines without affecting the signal amplitudes in comparison with WT (Figs 3e, S3a,b). We then crossed *isi1‐2* and *glr3.3a* plants. Upon wounding L8, without visible differences in the amplitudes of L13 SWPs measured in all the plants, *glr3.3a* mutation suppressed the effect of *isi1* on wound‐activated L13 SWPs in terms of duration (Fig. 3e), indicating that ISI1 functions through a GLR3.3‐dependent pathway. Then we examined phloem electrical signals using the established EPG approach that employs living aphids as electrodes (Salvador‐Recatala *et al*., 2014). L8 was wounded when the aphids were in the phloem‐feeding phase in the sieve elements of L13. As shown in Fig. 3f, compared with WT, *isi1‐2* increased both the amplitudes and durations of the depolarization signal in L13. Despite the prolonged duration in the mutant line, the expression of the defense marker gene *JAZ10* was induced to similar levels in both *isi1‐2* and WT plants (Fig. S3c). However, in our bioassay, *S. littoralis* gained less weight on *isi1‐2* relative to WT (Fig. 3g), indicating that *isi1‐2* mutants are more resistant to insect feeding than WT is. Collectively, our findings support the roles of ISI1 in wound‐associated responses.

### RFL residues in the GLR3.3 carboxy‐terminal tail interact with ISI1

**Fig. 4:**
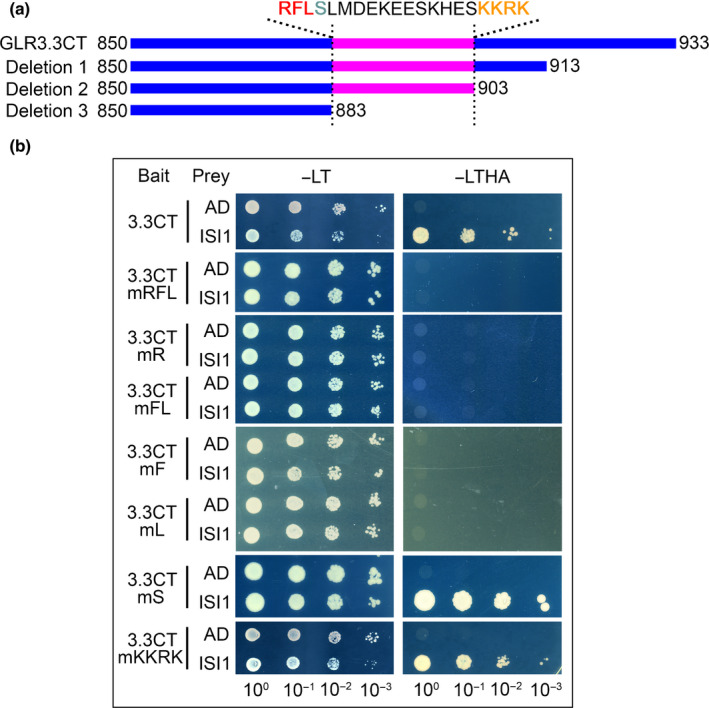
RFL residues in the GLUTAMATE RECEPTOR‐LIKE (GLR)3.3 carboxy‐terminal tail (C‐tail) are required for its interaction with IMPAIRED SUCROSE INDUCTION 1 (ISI1). (a) Schematic diagram showing serial deletions of GLR3.3 C‐tail that were used for mapping interacting sites. Amino acid sequences from position 884 to position 903 are presented. (b) Mutations in RFL residues (red in (a)), but not S (cyan in (a)) or KKRK (orange in (a)), abolish its interaction with ISI1 in yeast. GLR3.3 C‐tail carrying mutations in single or combinational RFL residues, S or KKRK, were cotransformed with ISI1 into yeast cells to test the interactions. −LT, Leu‐Trp‐; −LTHA, Leu‐Trp‐His‐Ade‐.

Next, in an attempt to identify the binding sites of ISI1 and GLR3.3CT, we constructed three serially deleted versions of GLR3.3 C‐tail (3.3CT) as baits (Fig. 4a) and tested their interactions with ISI1 in Y2H assays. In contrast to deletion 1 and deletion 2, which were both able to bind ISI1, the shorter 3.3CT truncation (deletion 3) lost its interaction with ISI1 (Fig. S4a). To further narrow down the sites, we performed site‐directed mutagenesis in the region of amino acids 883–903 and created different baits carrying 3.3CT point mutations. Intriguingly, among all the mutated C‐tail variants (Figs 4b, S4a), only those carrying mutations in the three amino acids Arg884 (R884), Phe885 (F885), and Leu886 (L886) (mRFL, mR, mFL, mF and mL) impaired the interaction with ISI1. A mutation in Ser887 (mS) next to RFL, or in a combination of 4 aa Lys900 (K900)/Lys901 (K901)/Arg902 (R902)/Lys903 (K903) (mKKRK) did not affect their interactions (Figs 4b, S4a). In the case of ISI1, none of the truncations we generated was capable of interacting with the GLR3.3 C‐tail, suggesting that full‐length ISI1 is required for its binding to GLR3.3 (Fig. S4b,c). Finally, we confirmed the expression of proteins in the yeast combinations with impaired interactions (Fig. S4d–f).

### Mutation in RFL residues abolishes GLR3.3 function in wound signaling

**Fig. 5:**
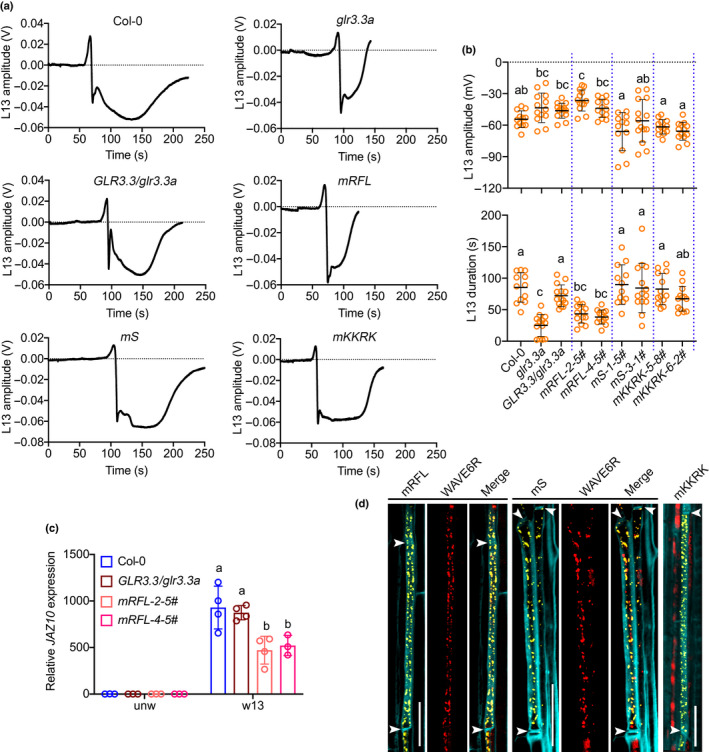
Mutation in RFL residues abolishes GLUTAMATE RECEPTOR‐LIKE (GLR)3.3 function in wound signaling without affecting its subcellular localization in Arabidopsis. (a) Exampled traces of leaf 13 (L13) slow wave potentials (SWPs) measured from GLR3.3 variant plants. (b) Quantitative analysis of amplitudes and durations of SWPs recorded on leaf 13 after wounding leaf 8. The yellow circles represent individual measurements. *n* = 9–15. Two independent lines for different GLR3.3 carboxy‐terminal tail (C‐tail) variants were measured in comparison with wild‐type, *glr3.3a* mutants, and *glr3.3a* complemented plants (*GLR3.3/glr3.3a*). The horizontal bars indicate the mean values. Error bars show SD. Letters represent significant differences (*P* < 0.05) after one‐way ANOVA. Blue dashed lines were introduced for better visualization of the differences between genotypes. (c) *JAZ10* expression in distal leaves 13 in comparison with unwounded leaves. mRFL variant was analyzed compared with control plants. Data shown are means ± SD. Colored points indicate different biological replicates. *n* = 3–4. The different letters indicate significant differences (*P* < 0.01) after two‐way ANOVA. (d) Subcellular colocalization of GLR3.3 C‐tail variants with the endoplasmic reticulum (ER) marker in the crossed plants. Yellow represents mVENUS signals from the C‐tail variants. Red indicates ER signals in the WAVE6R lines. Cyan marks the outlines of the cells. Arrowheads show the positions of the sieve plates. Bar, 20 μm.

Given that RFL residues in the GLR3.3 C‐tail are essential for its interaction with ISI1, we next sought to determine if these three amino acids were important for GLR3.3 biological function. We generated complementary plants that expressed GLR3.3 with different point mutations: RFL (*mRFL‐2‐5#* and *mRFL‐4‐5#*), Ser887 (*mS‐1‐5#* and *mS‐3‐1#*), and KKRK combination (*mKKRK‐5‐8#* and *mKKRK‐6‐2#*). Neither Ser887 nor KKRK is involved in the interaction with ISI (Fig. 4b). The aforementioned GLR3.3 C‐tail derivatives were individually fused with an mVENUS tag and transformed into *glr3.3a* mutant backgrounds. Surface potentials were measured in both the wounded L8s and the connecting L13s from all the plant lines. Variable L8 signals were detected in different genotypes (Fig. S5). In terms of the L13 signals, as shown in the example traces (Fig. 5a), the SWP trace of *mRFL* plants was similar to that of the *glr3.3a* mutant. However, all the other variants showed similar patterns compared with WT and the complemented *GLR3.3/glr3.3a* plants. We then quantified the amplitudes and durations of the L13 SWPs from all the plants. The amplitudes of the signal varied slightly among all the lines measured (Fig. 5b, upper panel). However, compared with WT and the *GLR3.3/glr3.3a* plants, plants carrying RFL mutations were incapable of rescuing the short‐duration SWP detected in the *glr3.3a* mutant. By contrast, the *mS* and the *mKKRK* plants all complemented the electrical signal phenotype of *glr3.3a* (Fig. 5b, lower panel). Next, wound‐induced *JAZ10* expression in the systemic leaves (L13) of *mRFL* plants that have compromised electrical signals was studied. Consistently, *JAZ10* induction in distal L13 upon wounding L8 was attenuated in *mRFL* plants (Fig. 5c). The different capacities of the GLR3.3 variants in rescuing the *glr3.3a* electrical signaling phenotypes promoted us to test if the subcellular localization of the proteins was altered. We separately crossed the *mRFL* plants (impaired L13 SWPs) and the *mS* plants (WT‐like L13 SWPs) to the WAVE6R plants expressing an ER marker protein (Geldner *et al*., 2009). Consistent with the published subcellular localization of the WT GLR3.3 protein (Nguyen *et al*., 2018), both variant proteins overlapped largely with the ER marker in the sieve elements (Fig. 5d). Moreover, the subcellular localization of another GLR3.3 variant in the *mKKRK* plants also showed a similar pattern in the sieve element (Fig. 5d).

### FL residues are functionally conserved in GLRs

**Fig. 6:**
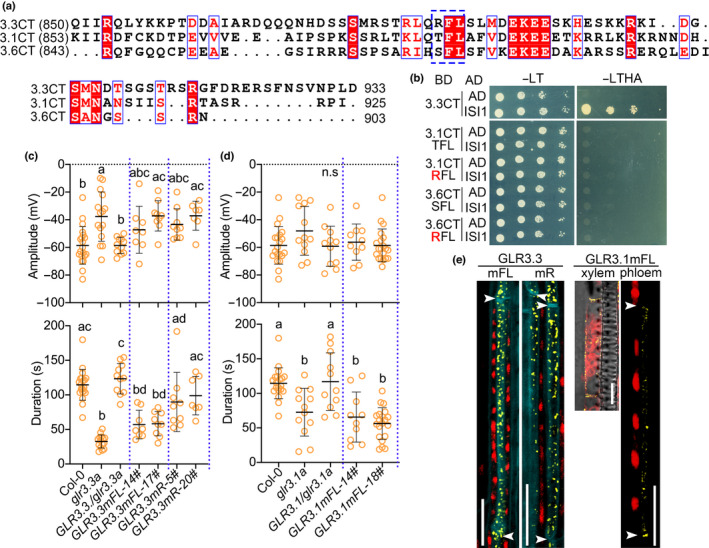
Conserved FL residues are required for GLUTAMATE RECEPTOR‐LIKEs (GLRs) function in electrical signaling. (a) Sequence alignments of the carboxy‐terminal tails (C‐tails) from three clade 3 GLRs in Arabidopsis. White characters shaded in the red boxes are strictly conserved. Red characters indicate similar residues. RFL, TFL, and SFL motifs in each C‐tail are marked by a blue dashed‐line box. (b) Mutations that convert TFL and SFL, respectively, in GLR3.1CT and GLR3.6CT to RFL do not promote their interaction with ISI1 in yeast two‐hybrid assay. −LT, Leu‐Trp‐; −LTHA, Leu‐Trp‐His‐Ade‐. (c, d) Surface potentials (amplitudes and durations) measured on the distal leaves 13 from different materials. In (c), GLR3.3 complementary lines carrying mR and mFL mutations were compared with the wild‐type (WT), *glr3.3a* mutant, and GLR3.3 complemented plants (*GLR3.3/glr3.3a*). In (d), GLR3.1 complementary lines carrying mFL mutation were compared with the WT, *glr3.1a* mutant, and GLR3.1 complemented plants (*GLR3.1/glr3.1a*). The WT samples are the same in (c, d). Two independent lines for each complementary construct were analyzed. The yellow circles represent individual measurements. *n* = 7–22. The horizontal bars indicate the mean values. Error bars show SD. Letters represent significant differences (*P* < 0.05) after one‐way ANOVA. ns, not significant. Blue dashed lines were introduced for better visualization of the differences between genotypes. (e) Subcellular localization of the GLR3.3 and GLR3.1 variant proteins fused to mVENUS. Yellow is signal from the fusion proteins of GLR3.3 and GLR3.1 variants. Red is Chl autofluorescence. Cyan marks outlines of the cells. Arrowheads indicate the positions of sieve plates. Images were taken with the same parameters. Bar, 10 μm in the panel showing GLR3.1 signal in a xylem contact cell. Bar, 20 μm in the other panels.

The impact of the RFL residues on GLR3.3 function promoted us to explore further the C‐tails of other GLRs involved in wound signaling. Interestingly, in the sequence alignment analysis of the C‐tails from GLR3.3, GLR3.1, and GLR3.6, we found FL residues were conserved among all three proteins. R residue in GLR3.3 was replaced by T in GLR3.1 and S in GLR3.6 (Fig. 6a). Notably, in the structural analysis of the three GLR proteins, the FL‐containing triresidue motifs were found to reside in the center of an α‐helix in their respective C‐tails (Fig. S6). We wondered if the differences of this site led to the failure in the interactions between the two GLRs and ISI1. TFL in GLR3.1 and SFL in GLR3.6 were then converted to RFLs in each GLR C‐tails. With proteins being properly expressed (Fig. S7), both GLRs carrying the converted RFL residues were still not able to bind ISI1 in yeast (Fig. 6b), suggesting a specific role of RFL sites in determining GLR3.3–ISI1 interaction.

Next, to investigate if the FL residues played a conserved role in GLR functions, we generated transgenic plants expressing GLR3.3mR, GLR3.3mFL, and GLR3.1mFL variants. Each variant protein was fused with an mVENUS tag and transformed into their respective mutant backgrounds. Then we measured wound‐induced leaf‐to‐leaf SWPs in the different lines. Wounding of L8 caused slightly variable SWPs (Fig. S8). More significantly, in the leaves distal to wounds (L13), both *GLR3.3mFL* and *GLR3.1mFL* variants failed to rescue the defects of their mutants in generating WT‐like electrical signals (Fig. 6c,d), implying conserved and indispensable functions of these two residues. By contrast, GLR3.3mR variant showed WT‐like responses (Figs 6c, S8f). Finally, we analyzed the subcellular localization of the variant proteins. Consistent with the reported subcellular localization of both proteins (Nguyen *et al*., 2018), none of the mutations affected their distributions in the cellular compartments (Fig. 6e).

## Discussion

Vertebrate iGluRs and plant GLRs are related. The C‐tails of iGluRs can play critical roles in receptor localization and function and can bind a variety of regulatory proteins that act to optimize their function. The importance of cytoplasmic C‐termini of vertebrate relatives of the plant GLRs is exemplified in synaptic iGluRs. Vertebrate iGluRs function not only as ligand‐gated ion channels but can also form large signaling complexes with diverse proteins. For example, vertebrate *N*‐methyl‐d‐aspartate (NMDA) receptors (NMDARs) in postsynaptic membranes are organized in signaling modules of > 1 MDa (Husi & Grant, 2001). In these complexes, the cytoplasmic C‐tails of the NMDARs bind directly and indirectly to multiple partners, including scaffold proteins, protein kinases, protein phosphatases, and transcriptional corepressors (Collins & Grant, 2007; Lau & Zukin, 2007; Hardingham, 2019). Furthermore, the C‐tail of vertebrate GluN1 can itself translocate to the nucleus to regulate synapse function (Zhou & Du, 2018). While many vertebrate NMDAR subunits, such as AMPA receptors, have long (> 500 aa) cytoplasmic C‐tails, other iGluRs from vertebrates with smaller C‐tails also form complexes with unrelated proteins (Hong *et al*., 2019). Despite the numerous studies on the C‐tails of iGluRs, the plant GLR C‐tails have never been investigated. Proteins with a potential to interact with the GLR3.3 C‐tail have not, to our knowledge, been identified. In this study, we first established the essential role of the GLR3.3 C‐tail. Considering the fact that the C‐tails of other GLRs can bind 14‑3‑3 scaffold proteins (Chang *et al*., 2009; Shin *et al*., 2011), we initiated a Y2H screen using the entire GLR3.3 C‐tail as a bait.

IMPA2 was confirmed to interact with the GLR3.3 C‐tail (Fig. S9a). However, the L13 SWPs were not affected in an *IMPA2* loss‐of‐function allele *impa2‐1* (SALK_017914, Fig. S9b,c). As the primary interactor, ISI1 is a plant‐specific protein that was reported as a positive regulator of the expression of sucrose‐inducible genes (Rook *et al*., 2006). The Rook *et al*. (2006) study also reported *ISI1* promoter activity in the phloem. However, how this expression pattern is linked to its role in regulating expression of sugar‐response genes remains unclear. Unlike IMPA2, loss‐of‐function *isi1* mutants showed elevated electrical activity in the leaves distal to wounds (Figs 3e, S3a,b), and this effect was dependent on the GLR3.3‐mediated pathway. Sieve‐element‐specific electrical signals were also prolonged in *isi1‐2* when measuring with aphid electrodes (Fig. 3f), consistent with the vasculature‐accumulated expression pattern of ISI1 protein (Fig. 3a–d). Moreover, *isi1*‐2 plants were more resistant to insect feeding (Fig. 3g), indicating that ISI1 plays a role in several aspects of wound responses. The response of *isi1* in wound‐induced leaf‐to‐leaf signaling is similar to that of *H* ^*+*^ *‐ATPase 1*, as reported previously (Kumari *et al*., 2019). In loss‐of‐function *aha1* proton pump alleles, a prolonged duration of SWPs is coupled with increased defense responses (Kumari *et al*., 2019). However, with respect to the induction of the jasmonate marker gene *JAZ10*, the transcripts were increased to similar levels in *isi1‐2* and WT plants upon wounding (Fig. S3c). We assume additional players are probably required together with ISI1 in the early induction of the canonical jasmonate pathway.

We further identified that a trio of residues (RFL) in the central region of the GLR3.3 C‐tail was required to bind ISI1 in yeast (Figs 4b, S4a). However, neither RFL nor *isi1* mutation altered the subcellular localization of major GLR3.3 pools (Figs 5d, S10), suggesting that other mechanisms may account for the impaired function of the *mRFL* plants (Fig. 5a–c). Given that ISI1 by itself does not have any apparent functional domain, it is considered less likely that ISI1 directly regulates GLR3.3 activity through RFL residues. We hypothesized that ISI1 possibly acts as a scaffold protein that links GLR3.3 to other regulators (Fig. S11). Examples of scaffold proteins binding to members of the glutamate receptor superfamily exist. For instance, in plants, the animal homologous cornichon proteins CNIH1 and CNIH4 interact with GLR3.3, controlling its membrane localization in pollen tubes (Wudick *et al*., 2018b). In the animal field, the C‐tails of GluN2 NMDAR subunits end in a small motif (xSxV) that binds scaffold proteins regulating receptor localization in postsynaptic membranes (Lau & Zukin, 2007; Bard & Groc, 2011). It will be interesting to isolate potential coregulators and test the scaffold hypothesis.

The C‐tails of the three GLRs contain several conserved sites, out of which ‘FL’ and ‘EKEE’ are the most representative (Fig. 6a). However, the latter was not involved in the interaction between GLR3.3CT and ISI1 in yeast. We thus evaluated the impact of ‘FL’ residues on GLR function in electrical signaling. We found that FL residues are functionally conserved in GLRs (Fig. 6c,d), implying a common regulatory mechanism through conserved sites in the C‐tails. However, the FL regulation of plant GLRs clearly represents a different strategy in comparison with iGluRs, as the subcellular localization of the proteins is not affected. *GLR3.3mR* plants generated similar L13 SWPs as in WT (Fig. 6c). This may suggest that the R residue is not crucial in determining GLR3.3 function but is likely more important in mediating the interaction with ISI1 in the proposed scaffolding model.

In summary, this work demonstrates that the short C‐tail of GLR3.3 is a key element in regulating its function in long‐distance signaling. Though considerable research has focused on the channel properties of GLR3.3, our results isolate key functional C‐tail residues and highlight from molecular and genetic perspectives the importance of the small C‐tail of GLR3.3 in its actions. The conserved FL residues across GLRs provide possibilities for future protein engineering aiming at designing stress‐resilient plants.

## Author contributions

QW and EEF conceived experiments; QW, SS and AK performed experiments; QW, SS and AK analyzed data; QW and EEF wrote the manuscript.

## Supporting information


**Fig. S1** Representative traces and quantitative analysis of slow wave potentials measured from the different genotypes as shown in Fig. 1b.
**Fig. S2** Characterization of Arabidopsis *isi1* alleles and subcellular localization of ISI1 fusion protein.
**Fig. S3** Wound‐induced slow wave potentials and *JAZ10* expression in Arabidopsis *isi1* mutants.
**Fig. S4** Domain mapping for GLR3.3‐ISI1 binding sites in yeast two‐hybrid assays.
**Fig. S5** Representative traces of electrical signals measured from the wounded leaves from genotypes as shown in Fig. 5a.
**Fig. S6** Structural prediction and analysis of three GLUTAMATE RECEPTOR‐LIKE proteins in Arabidopsis.
**Fig. S7** Western blotting analysis showing the expression of the bait and prey proteins in the yeast two‐hybrid assay corresponding to Fig. 6b.
**Fig. S8** Representative traces of electrical signals measured from both the wounded (black traces) and the distal (blue traces) leaves from genotypes as shown in Fig. 6(c,d).
**Fig. S9** IMPA2 interacts with GLR3.3 C‐tail, but does not affect leaf‐to‐leaf electrical signal propagation in Arabidopsis.
**Fig. S10** Subcellular localization of GLR3.3 is not affected by ISI1 inactivation in Arabidopsis.
**Fig. S11** A hypothetical model illustrating the roles of GLR3.3 C‐tail in leaf‐to‐leaf signaling.
**Table S1** Primer list for this study.
**Table S2** List of GLR3.3 C‐tail‐interacting candidates from yeast two‐hybrid screen.
Please note: Wiley Blackwell are not responsible for the content or functionality of any Supporting Information supplied by the authors. Any queries (other than missing material) should be directed to the *New Phytologist* Central Office.

## Data Availability

The data used to support the findings of this study are available upon request from the corresponding author.
