# Supplementary material for: The carboxy‐terminal tail of GLR3.3 is essential for wound‐response electrical signaling
Source: New Phytol. 2022 Sep 28;236(6):2189–201. doi: 10.1111/nph.18475 (PMC9828246; doi:10.1111/nph.18475)
Supplement: Supplementary file 1 — Fig. S1 Representative traces and quantitative analysis of slow wave potentials measured from the different genotypes as shown in Fig. 1b. Fig. S2 Characterization of Arabidopsis isi1 alleles and subcellular localization of ISI1 fusion protein. Fig. S3 Wound‐induced slow wave potentials and JAZ10 expression in Arabidopsis isi1 mutants. Fig. S4 Domain mapping for GLR3.3‐ISI1 binding sites in yeast two‐hybrid assays. Fig. S5 Representative traces of electrical signals measured from the wounded leaves from genotypes as shown in Fig. 5a. Fig. S6 Structural prediction and analysis of three GLUTAMATE RECEPTOR‐LIKE proteins in Arabidopsis. Fig. S7 Western blotting analysis showing the expression of the bait and prey proteins in the yeast two‐hybrid assay corresponding to Fig. 6b. Fig. S8 Representative traces of electrical signals measured from both the wounded (black traces) and the distal (blue traces) leaves from genotypes as shown in Fig. 6(c,d). Fig. S9 IMPA2 interacts with GLR3.3 C‐tail, but does not affect leaf‐to‐leaf electrical signal propagation in Arabidopsis. Fig. S10 Subcellular localization of GLR3.3 is not affected by ISI1 inactivation in Arabidopsis. Fig. S11 A hypothetical model illustrating the roles of GLR3.3 C‐tail in leaf‐to‐leaf signaling. Table S1 Primer list for this study. Table S2 List of GLR3.3 C‐tail‐interacting candidates from yeast two‐hybrid screen. Please note: Wiley Blackwell are not responsible for the content or functionality of any Supporting Information supplied by the authors. Any queries (other than missing material) should be directed to the New Phytologist Central Office. [file NPH-236-2189-s001.pdf]

New Phytologist

New Phytologist Supporting Information

Article title: The carboxy-terminal tail of GLR3.3 is essential for wound-response electrical signaling

Authors: Qian Wu, Stéphanie Stolz, Archana Kumari, Edward E. Farmer

Article acceptance date: 27 August 2022

The following Supporting Information is available for this article:

Fig. S1 Representative traces and quantitative analysis of SWPs measured from the different genotypes as shown in Fig. 1b.

Fig. S2 Characterization of Arabidopsis *isi1* alleles and subcellular localization of ISI1 fusion protein.

Fig. S3 Wound-induced SWPs and *JAZ10* expression in Arabidopsis *isi1* mutants.

Fig. S4 Domain mapping for GLR3.3-ISI1 binding sites in Y2H assays.

Fig. S5 Representative traces of electrical signals measured from the wounded leaves from genotypes as shown in Fig. 5a.

Fig. S6 Structural prediction and analysis of three GLR proteins in Arabidopsis.

Fig. S7 Western blotting analysis showing the expression of the bait and prey proteins in the Y2H assay corresponding to Fig. 6b.

Fig. S8 Representative traces of electrical signals measured from both the wounded (black traces) and the distal (blue traces) leaves from genotypes as shown in Fig. 6(c, d).

Fig. S9 IMPA2 interacts with GLR3.3 C-tail, but does not affect leaf-to-leaf electrical signal propagation in Arabidopsis.

Fig. S10 Subcellular localization of GLR3.3 is not affected by ISI1 inactivation in Arabidopsis.

Fig. S11 A hypothetical model illustrating the roles of GLR3.3 C-tail in leaf-to-leaf signaling.

Table S1 Primer list in this study.

Table S2 List of GLR3.3 C-tail-interacting candidates from Y2H screen.

New Phytologist

Fig. S1 Representative traces and quantitative analysis of SWPs measured from the different genotypes as shown in Fig. 1b. (a) Representative traces of L8 and L13 SWPs recorded from genotypes as shown in Fig. 1b. Red arrowheads indicate the time points when wounding was applied to leaves 8. (b) Amplitudes and durations of the surface potentials measured on the wounded leaves (L8) of Arabidopsis. The yellow circles represent individual measurements. The horizontal bars indicate the mean values. Error bars show S.D. The different letters indicate significant differences after one-way ANOVA.

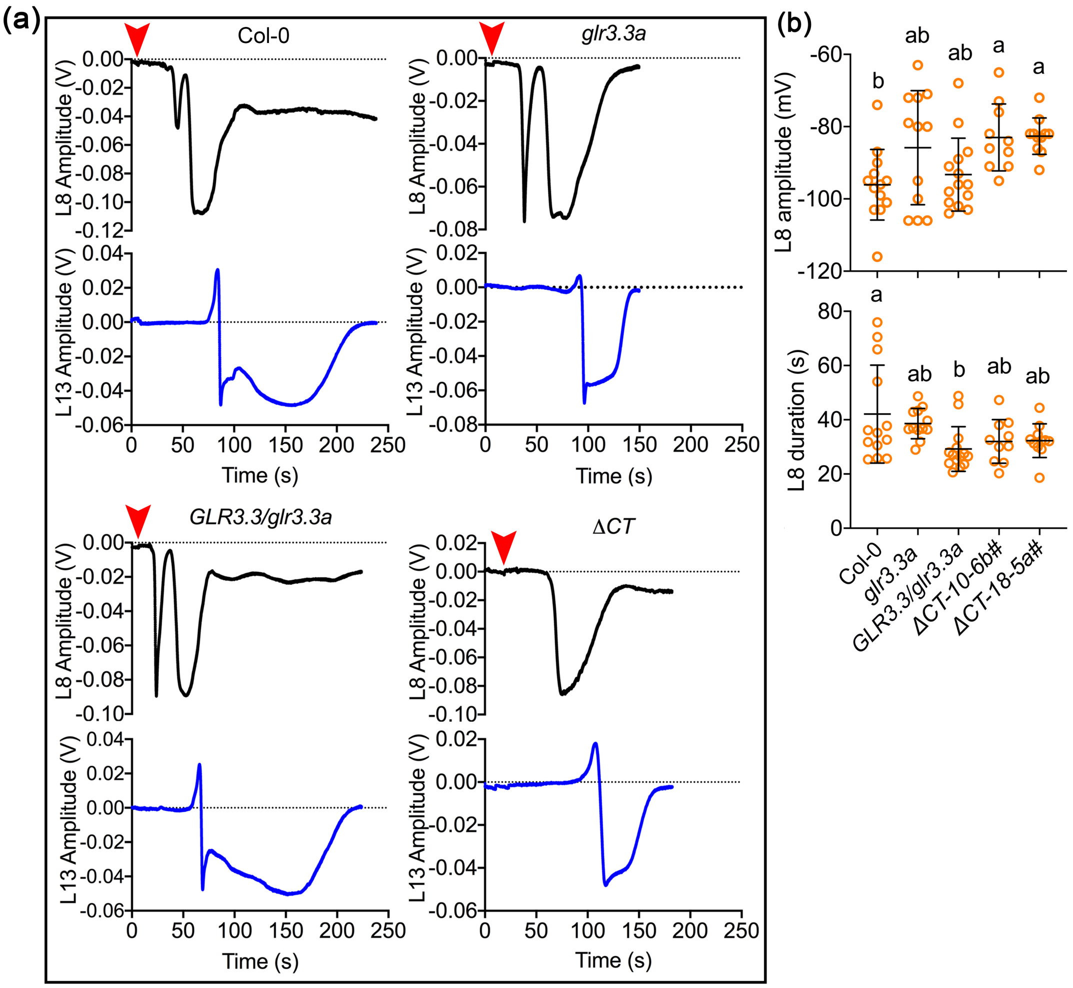

Fig. S2 Characterization of Arabidopsis *isi1* alleles and subcellular localization of ISI1 fusion protein. (a) The *ISI1* gene model. The positions of the T-DNAs from two *isi1* alleles are shown. (b) *ISI1* transcript levels in unwounded leaves from wild type and *isi* mutants. P1 and P2 in (a, b) indicate the regions that are amplified. Circles represent individual values. Data shown are means ± SD. n=3. (c) Subcellular localization of ISI1-mCherry fusion protein in the vascular tissue. Red signal represents mCherry fluorescence. Green color reflects chloroplast autofluorescence. DAPI staining (blue) marks the position of the nucleus in the companion cell. Signals from the above channels were merged together. Bar=10 μm. The dotted line marks the outline of a companion cell that is featured by the aligned chloroplast in the cell.

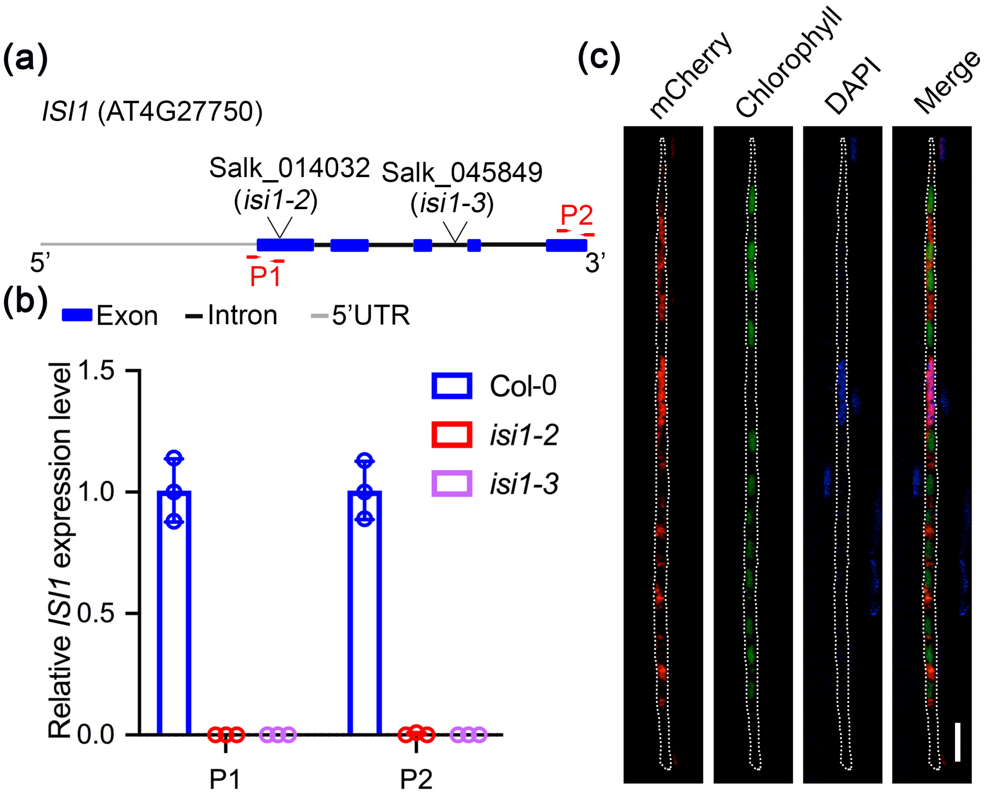

Fig. S3 Wound-induced SWPs and *JAZ10* expression in Arabidopsis *isi1* mutants. (a) Exampled traces of L8 and L13 SWPs recorded from WT and *isi1* mutants. Red arrowheads indicate the time points when wounding was applied to leaves 8. (b) Electrical signals (amplitudes and durations) recorded on the distal leaves 13 of *isi1-3* mutants compared to WT. The colored circles represent individual measurements. The horizontal bars indicate the mean values. Error bars show S.D. (c) Expressions of the jasmonate response gene *JAZ10* in distal leaf 13 of *isi1-2* mutants compared to WT after wounding leaf 8. The colored circles represent independent biological replicates. n=5. Error bars indicate S.D. *p* values were calculated with two-tailed Student's *t*-tests. n.s, not significant.

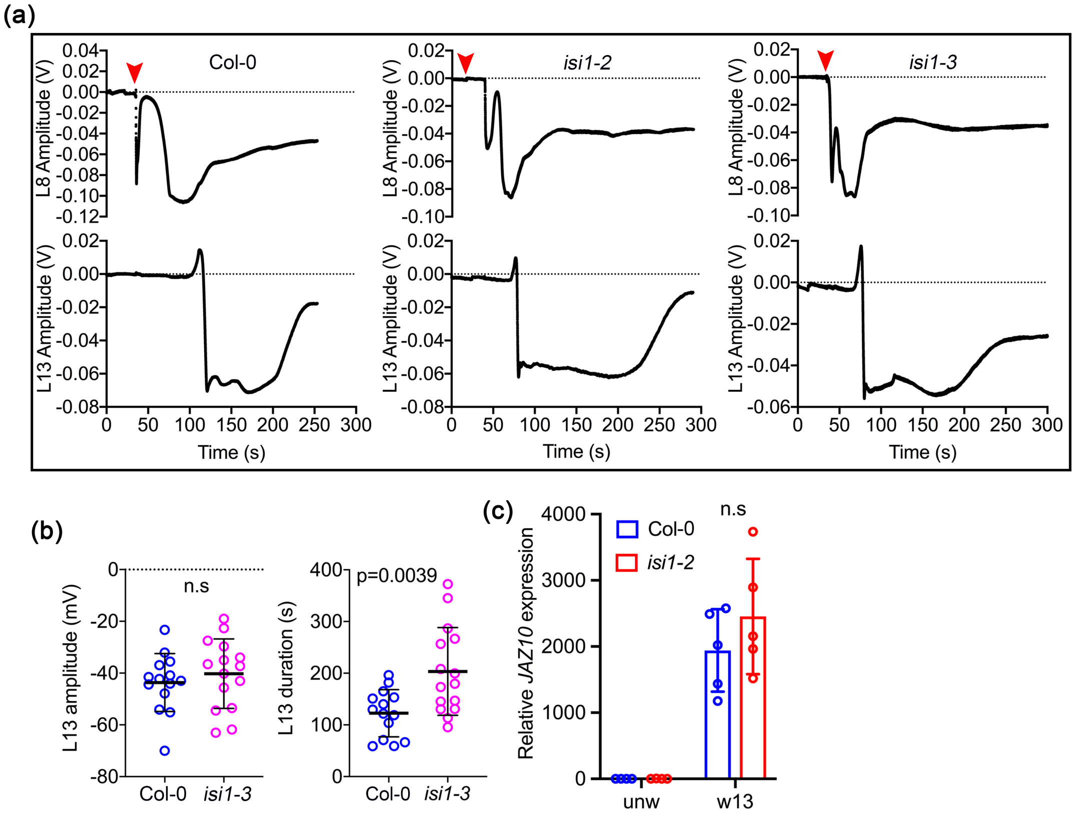

Fig. S4 Domain mapping for GLR3.3-ISI1 binding sites in Y2H assays. (a) ISI1 interaction with GLR3.3 C-tail (CT) variants. Images were taken after 3 or 5 days for yeast groups that were grown on Leu-Trp- (-LT)/Yeast Nitrogen Base (YNB) or Leu-Trp-His-Ade (-LTHA)/YNB medium, respectively. GLR3.3 CT and its mutants were co-expressed with empty AD (Activating Domain) vectors as negative controls. All the GLR3.3 C-tail variants (deletions and point mutations) were generated by PCRs using overlapping mutagenic primers and Binding Domain (BD)-3.3CT plasmid as template. (b) Schematic model for ISI1 protein. Residues from 136 to 239 in ISI1 were predicted by Pfam database to have a homology to Cell Division Control protein 14 (CDC14) in fission yeast. (c) Interaction analysis for the entire GLR3.3 C-tail with different ISI1 truncations. Empty BD vectors co-transformed with ISI1 truncated proteins served as negative controls. (d-f) Western blotting analysis showing the expression of the bait and prey proteins in the Y2H assay corresponding to Fig. 4b. Bait and prey proteins were detected with α-Myc and α-HA antibodies, respectively.

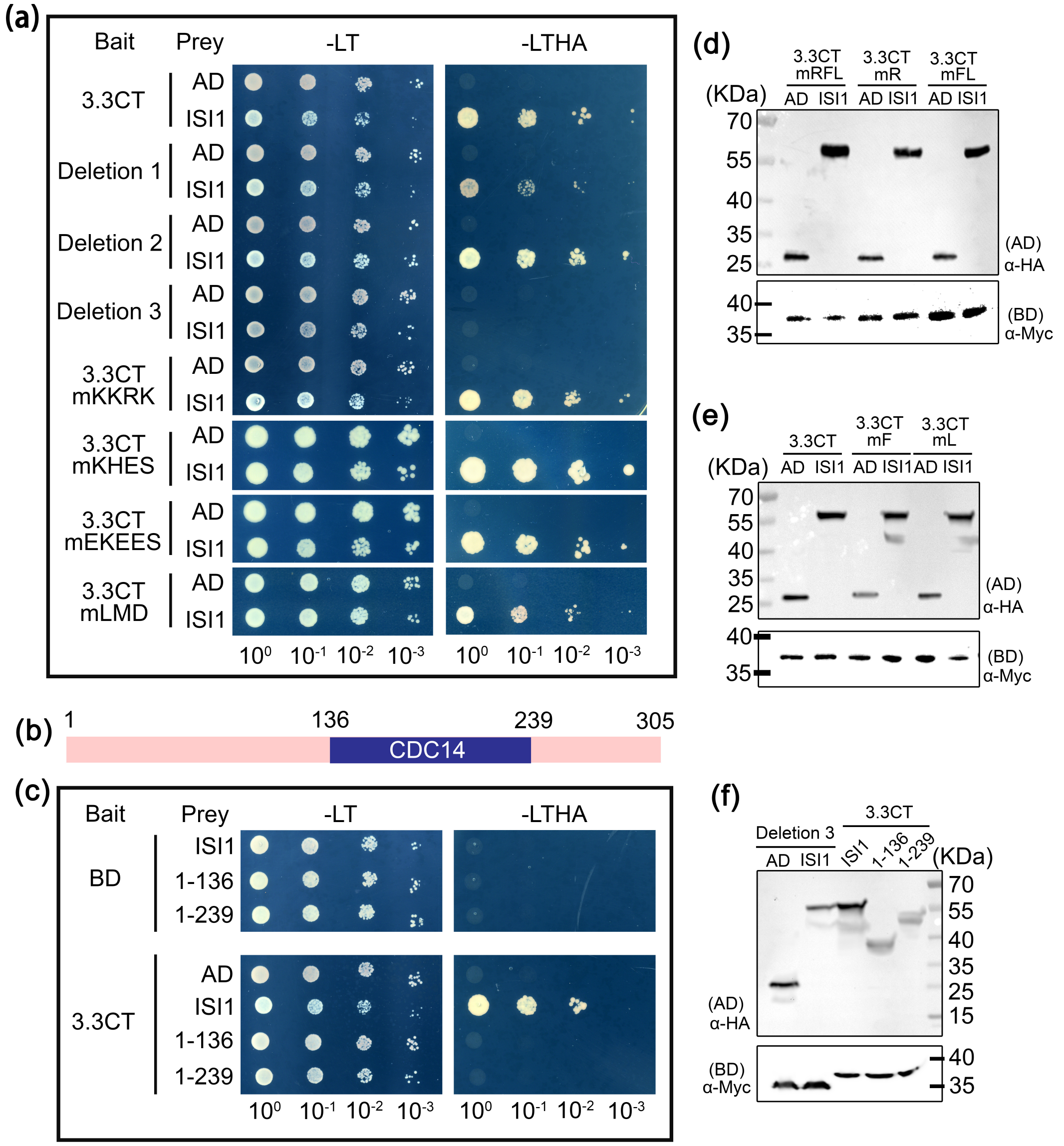
(a)
Bait
Prey
-LT
-LTHA
3.3CT
Deletion 1
Deletion 2
Deletion 3
3.3CT mKKRK
3.3CT mKHES
3.3CT mEKEES
3.3CT mLMD
AD
ISI1
10^0 10^{-1} 10^{-2} 10^{-3}
(b)
1
136
239
305
CDC14
(c)
Bait
Prey
-LT
-LTHA
BD
3.3CT
AD
ISI1
1-136
1-239
10^0 10^{-1} 10^{-2} 10^{-3}
(d)
3.3CT mRFL
3.3CT mR
3.3CT mFL
(KDa)
AD ISI1 AD ISI1 AD ISI1
70
55
40
35
25
40
35
(AD) α-HA
(BD) α-Myc
(e)
3.3CT
3.3CT mF
3.3CT mL
(KDa)
AD ISI1 AD ISI1 AD ISI1
70
55
40
35
25
40
35
(AD) α-HA
(BD) α-Myc
(f)
Deletion 3
3.3CT
AD ISI1
ISI1
1-136
1-239
(KDa)
70
55
40
35
25
15
40
35
(AD) α-HA
(BD) α-Myc

Fig. S5 Representative traces of electrical signals measured from the wounded leaves from genotypes as shown in Fig. 5a. The genotypes are: Arabidopsis Col-0 (a), *glr3.3a* mutants (b), GLR3.3 complemented plants (*GLR3.3/glr3.3a*, c), *mRFL* variants (d), *mS* variants (e) and *mKKRK* variants (f). Red arrowheads indicate the moment when wounding was applied to leaves 8.

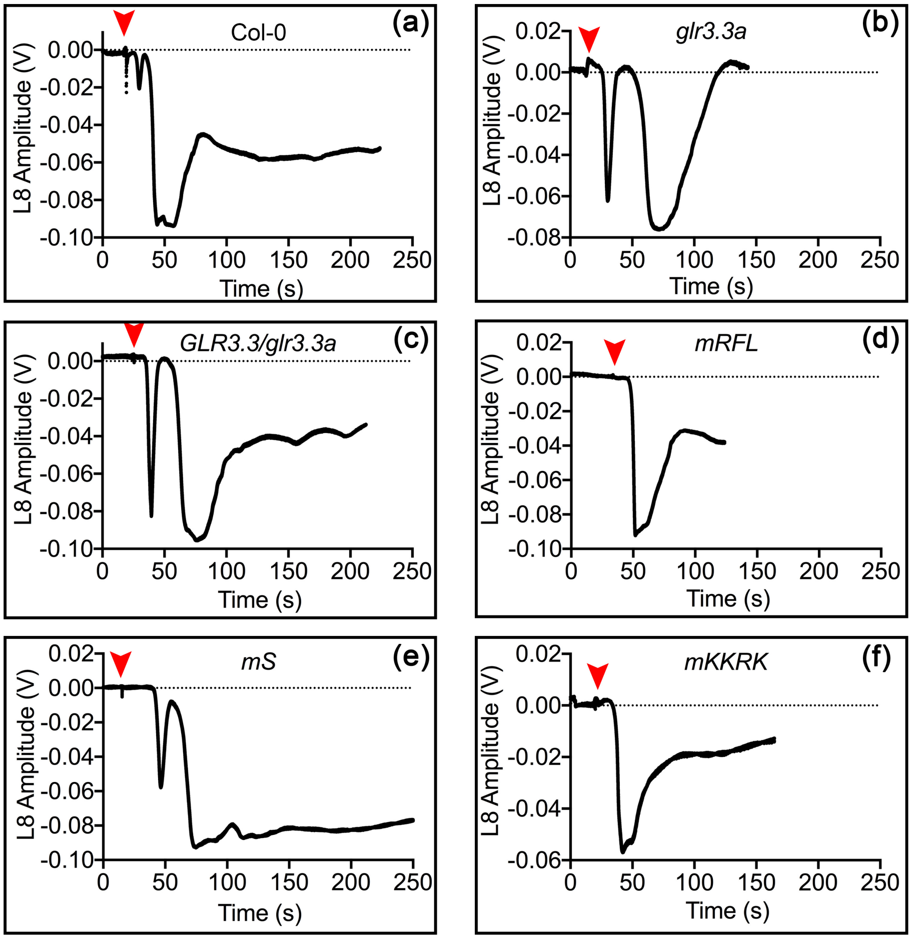

Fig. S6 Structural prediction and analysis of three GLR proteins in Arabidopsis. (a, d, f) Full-length GLR3.3, GLR3.1 and GLR3.6 protein structures were predicted with AlphaFold (AlphaFold Protein Structure Database). Red letters N and C indicate the amino-terminus and carboxyl-terminus of GLRs, respectively. GLR C-tails containing the triresidue motifs are highlighted by red circles. (b, e, g) Close-up view of partial GLR C-tails where the triresidue motifs are labeled. (c) Secondary structure and disorder prediction of GLR3.3 protein using Phyre2 (Protein Homology/analogY Recognition Engine V 2.0). A snapshot for the prediction result of region 841-900 amino acids is shown. RFL residues highlighted in a red dash-lined box are located in the centre of an α-helix structure. Confidence Key reveals levels of reliability for the prediction of different secondary structures.

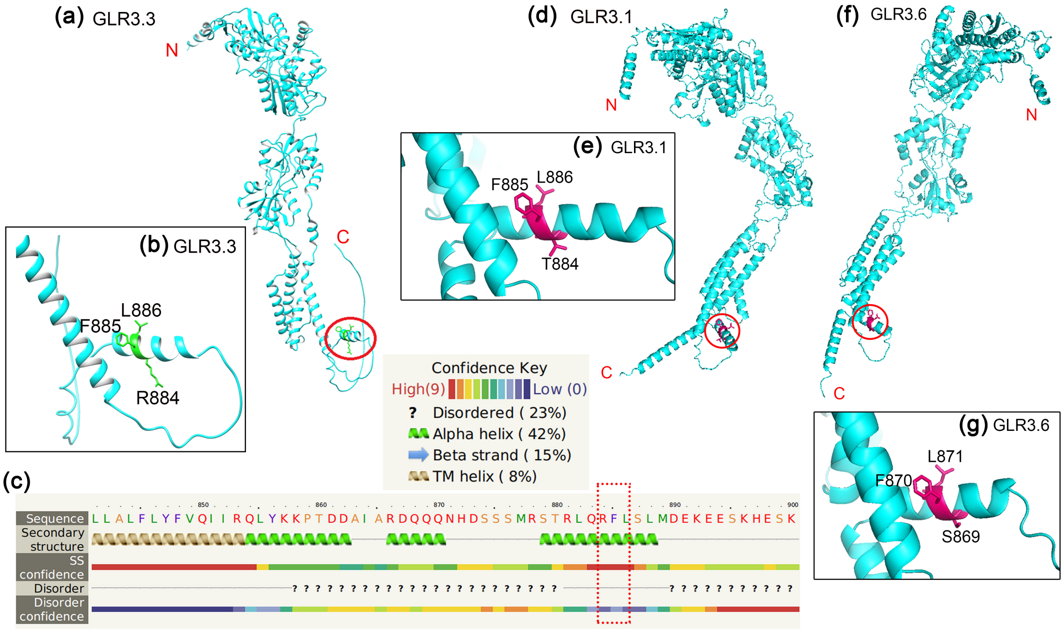

Fig. S7 Western blotting analysis showing the expression of the bait and prey proteins in the Y2H assay corresponding to Fig. 6b. Bait and prey proteins were detected with α-Myc and α-HA antibodies, respectively.

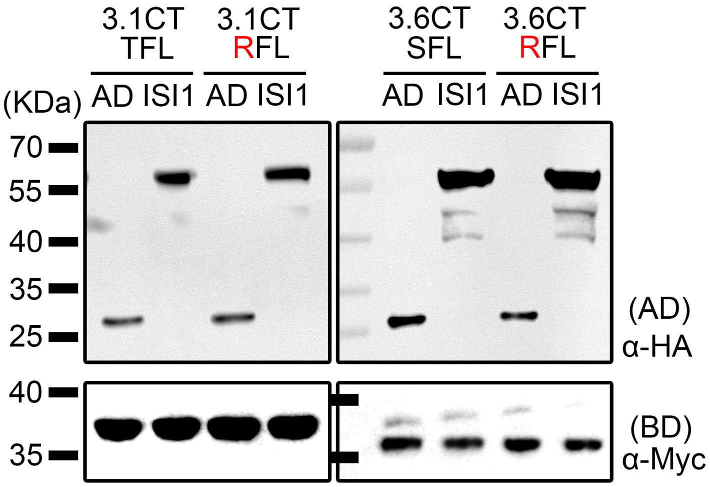

Fig. S8 Representative traces of electrical signals measured from both the wounded (black traces) and the distal (blue traces) leaves from genotypes as shown in Fig 6 (c, d). The genotypes include: Arabidopsis Col-0 (a), GLR3.3 complemented plants (*GLR3.3/glr3.3a*, b), GLR3.1 complemented plants (*GLR3.1/glr3.1a*, c), *glr3.3a* mutants (d), *GLR3.3mFL* variants (e), *GLR3.3mR* variants (f), *glr3.1a* mutants (g) and *GLR3.1mFL* variants (h). Red arrowheads indicate the time points when wounding was applied to leaves 8.

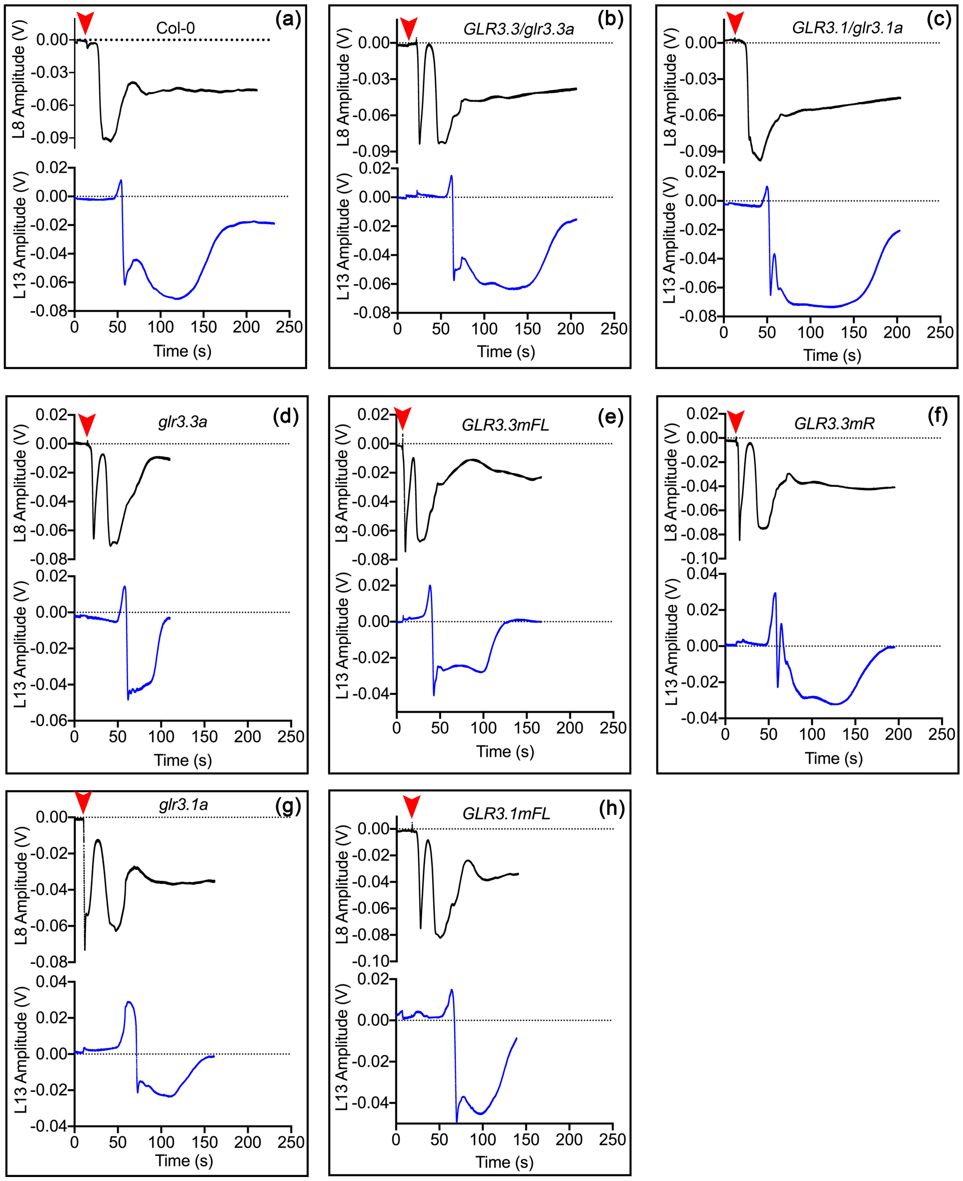

Fig. S9 IMPA2 interacts with GLR3.3 C-tail, but does not affect leaf-to-leaf electrical signal propagation in Arabidopsis. (a) IMPA2 interacts with GLR3.3CT in Y2H assay. (b, c) Amplitudes and durations of the electrical signals measured on the distal leaves of WT and *impa2-1* mutants upon wounding. The yellow circles represent individual measurements. n=12. The horizontal bars indicate the mean values. Error bars show S.D. n.s, not significant.

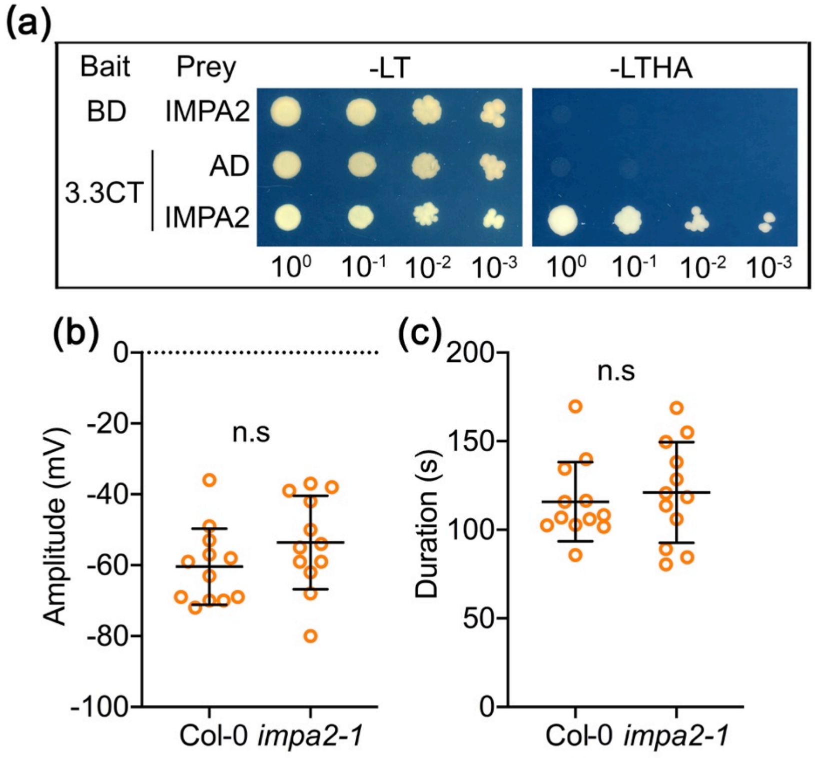

Fig. S10 Subcellular localization of GLR3.3 is not affected by ISI1 inactivation in Arabidopsis. GLR3.3-mVENUS localization in the *isi1-2* background. To study the impact of *ISI1* mutation on GLR3.3 intracellular distribution, the reported *glr3.3* complemented plants (GLR3.3g-mVENUS) by Nguyen et al. was crossed with *isi1-2*. mVENUS fluorescence was detected by confocal microscopy. Asterisks indicate the positions of sieve plates. Bars in all the images =10 µm. Images were taken under the same parameters in each panel.

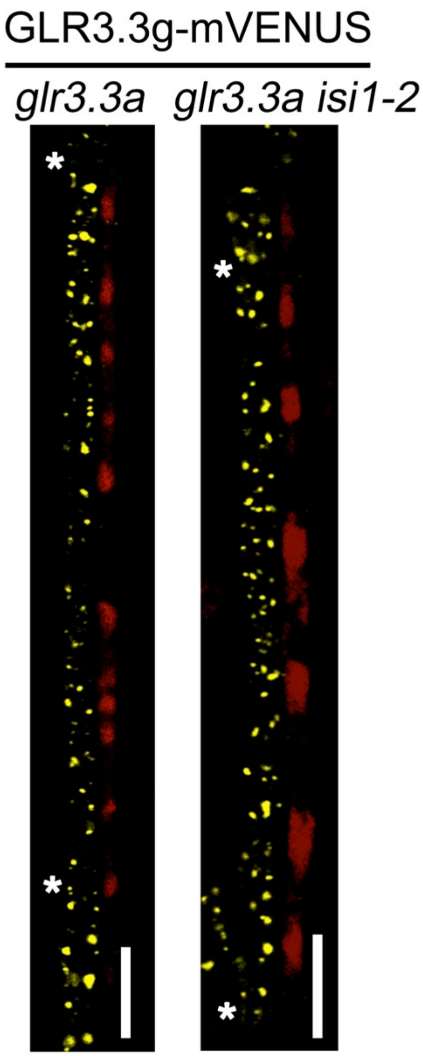

Fig. S11 A hypothetical model illustrating the roles of GLR3.3 C-tail in leaf-to-leaf signaling. Upon crushing leaf 8 (L8) of a 5-week-old Arabidopsis plant, SWPs were transmitted through the vasculature (as indicated by the red dashed line with arrowheads) to the connected leaf 13 (L13). The propagation of SWPs requires GLR3.3 that is predicted to be a multimembrane spanning protein with a short cytoplasmic C-tail. In our work, we identified a new binding protein called ISI1 that interacts with the RFL residues of GLR3.3 C-tail. We propose ISI may recruit other co-regulators to modulate GLR3.3 function in wound signaling.

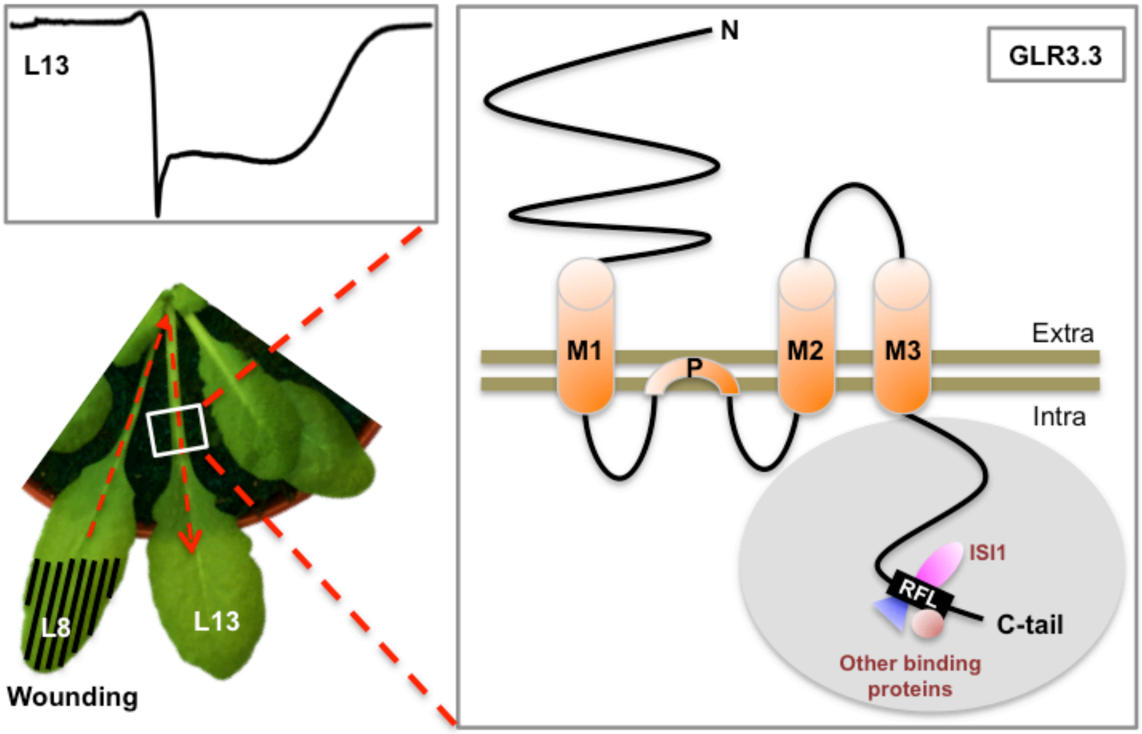

Table S1 Primer list in this study.

Primers used for genotyping T-DNA mutants	
Primer name	Primer sequence (5'-3')
ISI1-2-RP<br>ISI1-2-LP	CATCAGCCATTTTTCCAGTTC<br>CACCTCTCACTCTGATTTGGC
ISI1-3-RP<br>ISI1-3-LP	CTCCATCCTCAGAGCACTGTC<br>GGACACGTAAATGGGAAGGAT
IMPA2-1-RP<br>IMPA2-1-LP	CATCAAAAAGGAAGCTTGCTG<br>CCAGACTAAGTAAGGCACCCC

Primers used for cloning		
Primer name	Primer sequence (5'-3')	Vectors
ISI1(Pro+Gene) -F<br>ISI1(Pro+Gene) –R	ggaattcGGTACCATGTATCTGAAGAGACCGATATGG<br>ttcccccccgggagtacaagtcaagagactcgagca	pUC57
GLR3.3-S-Kpn1infusion<br>GLR3.3-A-Sal1infusion	CGGGGGACGAGCTCGGTACCATGAAGCAACTCTGGACTTT<br>ACGAGATCTGGTCGACGTCTAATGGATTTACCGAATT	pCAMBIA1300-nLUC
3.3CT-S-Kpn1<br>3.3CT-2A-Sal1	cggGGTACCATGCAGATCATCCGTCAGCTCT<br>gcGTCGACGTCTAATGGATTTACCGAATT	pCAMBIA1300-nLUC
ISI1-S-kpn 1<br>ISI1-A-Sal1	ggaattcGGTACCATGTATCTGAAGAGACCGATATGG<br>acgcGTCGACTTAGTACAAGTCAAGAGACTCGAGC	pCAMBIA1300-cLUC
3.3CT-S-EcoR1<br>3.3CT-A-Sal 1	cgGAATTCCAGATCATCCGTCAGCTCTATA<br>gcGTCGACTCAGTCTAATGGATTTACCGA	pGBKT7
3.6CT-S-EcoR1<br>3.6CT-A-Sal 1	cgGAATTCCGTCAGTTTGGACAGCAATG<br>gcGTCGACTTAGTTGCAGCGACTTGAACC	pGBKT7
3.1CT-S-EcoR1<br>3.1CT-A-Sal 1	cgGAATTCGTGCATAGCTTCTGGGGTATG<br>gcGTCGACTCATATGGGTCTTCTAGATGCAG	pGBKT7
ISI1-S-Nde 1<br>ISI1-A-Sal 1	ggaattcCATATGATGTATCTGAAGAGACCGATATGG<br>acgcGTCGACTTAGTACAAGTCAAGAGACTCGAGC	pGADT7-Rec
IMPA2-S-Nde 1<br>IMPA2-A-Sal 1	ggaattcCATATG ATGTCTTTTGAGACCTAACGCTA<br>acgcGTCGAC TCACTGGAAGTTGAATCCAC	pGADT7-Rec
GLR3.3$_{pro}$:GLR3.3ΔCT	gtatagaaaagttg ggtacc acccaaaccgcttattcttg	pUC57

-F<br>GLR3.3$_{pro}$:GLR3.3ΔCT<br>-R	tgtacaaacttgt cccggg aacaaagtataggaagag	

Primers used for site-directed mutagenesis

Primer name	Primer sequence (5'-3')
3.3CT-mKKRK-F<br>3.3CT-mKKRK-R	tcccgatgtatcgttcattgaaccatcgatcgctgccgccgcgctttcgtgcttggactcttctttatcatcc<br>ggatgagaaagaagagtccaagcacgaaagcgcggcggcagcgatcgatggttcaatgaacgatacatcggga
3.3CT-mS-F<br>3.3CT-mS-R	ctcgtttgcaaagattcttggctctcatggatgagaaagaa<br>ttctttctcatccatgagagccaagaatctttgcaaacgag
3.3CT-mRFL-F<br>3.3CT-mRFL-R	ctcttctttctcatccatgagagacgcggctgcttgcaaacgagtggagcgcatggag<br>cctccatgcgctccactcgtttgcaagcagccgcgtctctcatggatgagaaagaaga
3.3CT850-913-S<br>3.3CT850-913-A	tgaacgatacatcgggatgagtcgacctgcag<br>ctgcaggtcgactcatcccgatgtatcgttca
3.3CT850-903-S<br>3.3CT850-903-A	cgctgcaggtcgactcactttctcttcttgcttt<br>aaagcaagaagagaaagtgagtcgacctgcagcg
3.3CT850-883-S<br>3.3CT850-883-A	caggtcgactcattgcaaacgagtggagcgca<br>tgcgctccactcgtttgcaatgagtcgacctg
3.3CT-mLMD-S<br>3.3CT-mLMD-A	gcttggactcttctttctcagccgcggcagacaagaatctttgcaaacgagtggagc<br>gctccactcgtttgcaaagattcttgtctgccgcggctgagaaagaagagtccaagc
3.3CT-mEKEES-S<br>3.3CT-mEKEES-A	atctttctcttcttgctttcgtgcttggccgctgctgccgcatccatgagagacaagaatctttgcaa<br>ttgcaaagattcttgtctctcatggatgcggcagcagcggccaagcacgaaagcaagaagagaaagat
3.3CT-mKHES-S<br>3.3CT-mKHES-A	gttcattgaaccatcgatctttctcttcttggctgcggccgcggactcttctttctcatccatgagagacaag<br>cttgtctctcatggatgagaaagaagagtccgcggccgcagccaagaagagaaagatcgatggttcaatgaac
3.3CT-mR-S<br>3.3CT-mR-A	atccatgagagacaagaatgcttgcaaacgagtggagcgc<br>gcgctccactcgtttgcaagcattcttgtctctcatggat
3.3CT-mFL-S<br>3.3CT-mFL-A	cttctttctcatccatgagagacgcggctctttgcaaacgagtggagcgc<br>tgcgctccactcgtttgcaaagagccgcgtctctcatggatgagaaagaag
3.1CT-mFL-S<br>3.1CT-mFL-A	ccttttcatcaacaaatgctgcagcagtctgtagctttgttaaacgtgaagattttggtgaag<br>ccttcaccaaaatcttcacgtttaacaaagctacagactgctgcagcatttgttgatgaaaagg
ISI1-1-239-S<br>ISI1-1-239-A	tgcggagagttcttactataagtcgagctgcagatg<br>catctgcagctcgacttatagtaagaactctccgca
ISI1-1-136-S<br>ISI1-1-136-A	tcagtgtggagccgatgaagatataagtcgagctgc<br>gcagctcgacttatatcttcatcggctccacactga

Primers used for qPCR

Primer name	Primer sequence (5’-3’)
ISI1-RS1	GAATCCATCAGAATCGGAGACC
ISI1-RA1	CGGATGCGAAGGAAGGAGA
ISI1-RS2	CAATAGCGAGTGTAAATGAAGACAT
ISI1-RA2	TCTACCAGCCTGGATATGAAGC

Table S2 List of GLR3.3 C-tail-interacting candidates from Y2H screen.

Gene ID	Global PBS	Gene ID	Global PBS
AT1G30520	N/A	AT4G27750	A
AT3G11200	N/A	AT4G27750	A
AT4G04610	N/A	AT4G27750	A
AT4G38900	N/A	AT4G27750	A
AT4G38900	N/A	AT4G27750	A
AT4G38900	N/A	AT4G27750	A
AT4G38900	N/A	AT4G27750	A
AT4G38900	N/A	AT4G27750	A
AT1G43890	N/A	AT4G27750	A
AT1G43890	N/A	AT4G27750	A
AT5G35735	N/A	AT4G27750	A
AT5G20230	N/A	AT3G05970	N/A
AT1G29930	N/A	AT1G77590	D
AT1G29930	N/A	AT3G47470	N/A
AT5G19450	D	AT1G06220	D
AT1G20440	N/A	AT5G57040	N/A
AT5G20720	N/A	AT4G02150	N/A
AT1G09340	N/A	AT5G04910	D
AT2G27690	N/A	AT3G57560	N/A
AT4G34200	D	AT3G07780	N/A
AT3G56150	N/A	AT2G44490	N/A
AT2G39050	D	AT3G59060	E
AT5G61010	N/A	AT2G39730	N/A
AT2G25520	N/A	AT5G66160	N/A
AT2G37520	D	AT2G37340	N/A
AT4G12040	N/A	AT1G67870	N/A
AT1G63010	N/A	AT4G34450	N/A
AT5G03610	N/A	AT1G53230	D
AT1G52200	N/A	AT3G46090	N/A
AT1G02930	N/A	AT4G14410	D

AT1G02930	N/A	AT4G14410	D
AT3G06720	B	AT4G14410	D
AT3G06720	B	AT1G10585	N/A
AT3G06720	B	AT1G25550	N/A
AT4G16143	B	AT1G09270	N/A
AT4G16143	B	AT1G09270	N/A
AT4G16143	B	AT1G09270	N/A
AT4G16143	B	AT5G38410	N/A
AT4G16143	B		
AT4G27750	A		
AT4G27750	A		
AT4G27750	A		
AT4G27750	A		
AT4G27750	A		

Notes: The Predicted Biological Score (PBS) is a score that is automatically computed through algorithms. PBS categories represent the confidences for the interactions in the screen. A: Very high confidence in the interaction; B: High confidence in the interaction; D: Moderate confidence in the interaction; E: Interactions involving highly connected (or relatively highly connected) prey domains, warning of non-specific interaction. N/A: The PBS score cannot be attributed.
